# The Importance of ATM and ATR in *Physcomitrella patens* DNA Damage Repair, Development, and Gene Targeting

**DOI:** 10.3390/genes11070752

**Published:** 2020-07-06

**Authors:** Martin Martens, Ralf Horres, Edelgard Wendeler, Bernd Reiss

**Affiliations:** 1 Max-Planck-Institute for Plant Breeding Research, Plant DNA Recombination Group, Carl-von-Linne-Weg 10, D-50829 Cologne, Germany; mmartens1979@gmx.de (M.M.); Wendeler@mpipz.mpg.de (E.W.); 2 GenXPro GmbH, Altenhöferallee 3, D-60438 Frankfurt am Main, Germany; horres@genxpro.de

**Keywords:** *Physcomitrella patens*, DNA damage repair, double-strand break repair, phosphoinositide 3-kinase-related kinases, gene targeting, evolution, transcriptional control, development, DNA recombination

## Abstract

Coordinated by ataxia-telangiectasia-mutated (ATM) and ATM and Rad3-related (ATR), two highly conserved kinases, DNA damage repair ensures genome integrity and survival in all organisms. The *Arabidopsis thaliana* (*A. thaliana*) orthologues are well characterized and exhibit typical mammalian characteristics. We mutated the *Physcomitrella* *patens* (*P. patens*) *PpATM* and *PpATR* genes by deleting functionally important domains using gene targeting. Both mutants showed growth abnormalities, indicating that these genes, particularly *PpATR*, are important for normal vegetative development. ATR was also required for repair of both direct and replication-coupled double-strand breaks (DSBs) and dominated the transcriptional response to direct DSBs, whereas ATM was far less important, as shown by assays assessing resistance to DSB induction and SuperSAGE-based transcriptomics focused on DNA damage repair genes. These characteristics differed significantly from the *A. thaliana* genes but resembled those in yeast (*Saccharomyces cerevisiae*). *PpATR* was not important for gene targeting, pointing to differences in the regulation of gene targeting and direct DSB repair. Our analysis suggests that ATM and ATR functions can be substantially diverged between plants. The differences in ATM and ATR reflect the differences in DSB repair pathway choices between *A. thaliana* and *P. patens*, suggesting that they represent adaptations to different demands for the maintenance of genome stability.

## 1. Introduction

DNA damage caused by intrinsic and extrinsic factors constantly challenges genome stability [1]. Repair of DNA damage employs a complex network of interacting pathways, the DNA damage response (DDR), comprising DNA damage sensing, signal integration, and signal transduction processes that activate diverse DNA damage repair pathways and their connections to DNA replication and cell-cycle control [2,3,4,5]. DNA double-strand breaks (DSBs) represent a lethal form of DNA damage, as failure to repair such damage causes loss of genetic information and affects cellular viability. DSB repair occurs by two principal pathways, non-homologous end-joining (NHEJ) and homologous recombination (HR) [6,7], processes that differ in their requirement for a homologous template, their precision and speed of repair, and their dependence on the state of the cell-cycle [5,8,9]. Central components in regulating cell-cycle arrest and DSB repair in mammals are the phosphoinositide 3-kinase-related kinase (PIKK) proteins ataxia-telangiectasia-mutated (ATM), ATM and Rad3-related (ATR), and the DNA-dependent protein kinase catalytic subunit (PRKDC) [10], two of which, ATM and ATR, are evolutionarily conserved across all eukaryotes.

Ionizing radiation (IR) and radiomimetics such as bleomycin cause DSBs directly (direct DSBs) while the DNA damage caused by a number of other agents such as mitomycin C is converted to DSBs by replication (replication-coupled DSBs). In humans, *atm* mutants are markedly more sensitive to agents causing direct DSBs than *atr* mutants, and *atr*, but not *atm* mutants, are highly sensitive to agents introducing replication-coupled DSBs [8,9]. These resistance patterns suggest that ATM and ATR are specialized to different types of DSBs and cooperate to build the central hub in the human DDR [11,12]. *Arabidopsis thaliana atm* mutants are also highly sensitive to IR and methyl methanesulfonate (MMS), but not UV-B [13] and the radiomimetic bleomycin [14], indicating the importance of ATM in the repair of direct DSBs. *Atatr* mutants are less sensitive to IR [15] but are highly sensitive to agents inducing replication-coupled DSBs such as replication blocking agents (aphidicolin, UV-B, hydroxyurea) [15,16] and the inter-strand crosslinking agent mitomycin C [14]. These resistance patterns suggest a comparable specialization of ATM and ATR functions in plants [17]. In addition, ATM plays a leading role in the control of the transcriptional response to direct DSBs in *A. thaliana*, whereas ATR is of minor importance [15,18].

*Physcomitrella patens* (*P. patens*) is the model moss representing the bryophytes in the land plant lineage, the earliest diverging clade among the plants colonizing the land [19,20,21]. In addition, the high efficiencies of gene targeting in *P. patens* [22], a process based on homology-mediated integration of transforming DNA, are unique in the land plant lineage and contribute to the importance of this organism as a model. Previous analyses of core recombination genes in HR (*RAD51, RAD51* paralogue *B*) demonstrated that HR is the preferred repair pathway for direct DSBs in *P. patens*, although NHEJ contributes [23], whereas *A. thaliana* mainly relies on NHEJ [24,25,26]. In addtion, analyses of additional recombination genes (*MRE11–RAD50–NBS1* (MRN)) complex, *MSH2*, and *RECQ*) [27,28,29] showed the importance of this process for DNA damage repair in *P. patens*. Analysis of the genes directly involved in HR (*RAD51, RAD51* paralogue *B*) showed that these are also essential for gene targeting [25,26,30] and suggested that this process is solely based on RAD51-mediated recombination in *P. patens*, although this dependence is restricted on gene targeting in the absence of DSB induction [31] Together with the analyses of other genes [27,28,29], these data suggest a tight connection between DSB repair and gene targeting in *P. patens*. 

To analyze genes not directly involved in recombination, we mutated the *P. patens ATM* and *ATR* genes by gene targeting. Characterization of the corresponding mutants and a double mutant obtained by crossing showed that both mutations caused developmental aberrations in *P. patens*. The *atm* mutant in *P. patens* was also only barely sensitive to bleomycin, and the transcriptional control of major DNA damage repair genes was only marginally dependent on ATM. Rather, both the transcriptional response induced by bleomycin and resistance to such damage was largely dependent on ATR. By contrast, neither ATR nor ATM alone was important for gene targeting, but both together they were. Our data highlight the importance of regulatory components in these processes and reveal fundamental differences in the regulation of DSB repair and gene targeting as well as in ATM and ATR functions between *A. thaliana* and *P. patens.*

## 2. Materials and Methods 

### 2.1. Identification and Characterisation of PpATM and PpATR Genes 

Genes were originally identified by homology searches using TBLASTN with the entire *A. thaliana* protein sequences as query in the initial v1.1 gene model DNA sequences of the *P. patens* genome [32]. Using total protonemal RNA as starting material and gene specific primers, the corresponding complementary DNA (cDNAs) were amplified by PCR, cloned, and Sanger sequenced. Finally, the extreme 5′ and 3′ ends of the transcripts were determined by 5′ and 3′ rapid amplification of cDNA ends (RACE) (5′/3′ RACE Kit, Roche) using the same RNA as starting material and gene-specific primers located at terminal exons: ATM_Ex3_1_rev (CCA GAA CGT CCA TTA GTA TGC AAT CG), ATR_Ex1_2_rev (CCT AAG ATT GGT AAC ACT GCA TGG C), ATM_Ex73_1_fwd (CTG CTG ACA TGG ACA ACT CGG AG), and ATR_EX22_1_fwd (TCG AAA TCC TAG CCT CGC TGC). Gaps were filled by long template PCR using gene-specific primers, the entire cDNA amplified using primers at the extreme ends of the untranslated regions (UTRs), and PCR products cloned in pGEM-t-easy (Invitrogen). The inserts were Sanger sequenced and final sequences were corrected for PCR and sequencing errors using the genomic sequence as a template.

**Figure 1:**
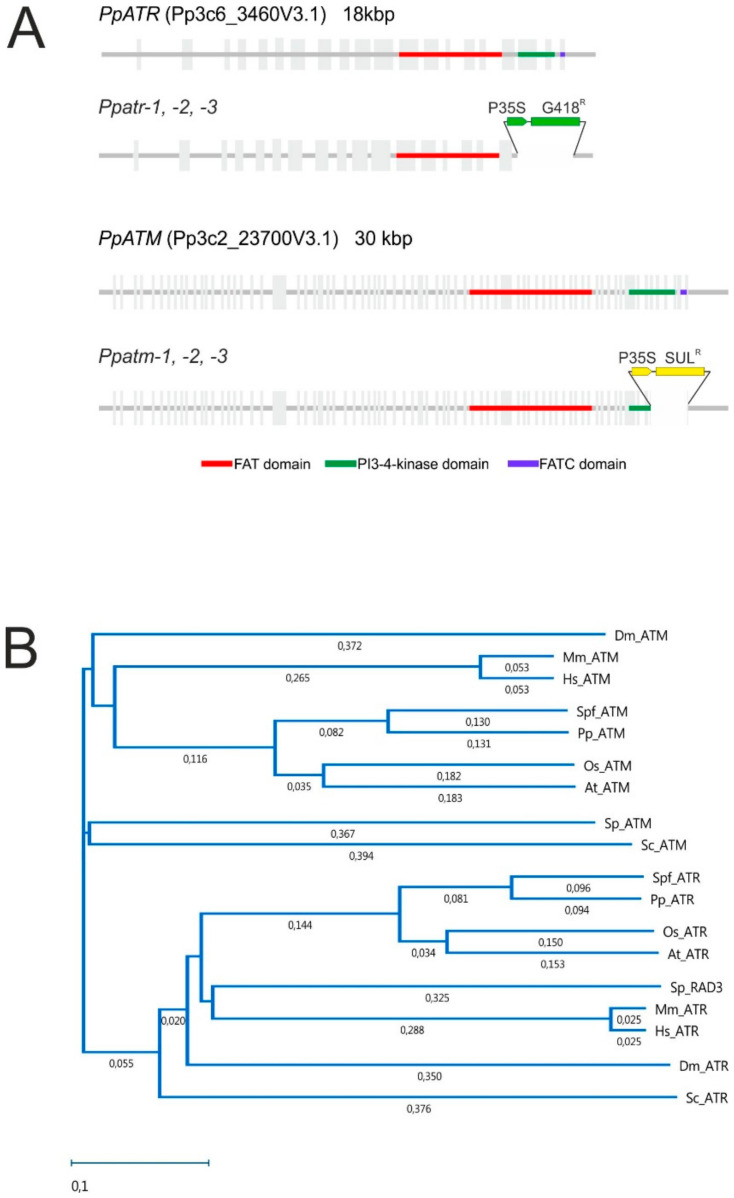
Phylogenetic analysis, gene structure, and targeted replacements. (**A**) The structure of *PpATM* and *PpATR* genes is shown with exons indicated in light gray and the conserved FAT, PI3-4 kinase, and FAT-C domains in color. The domain replacements obtained by gene targeting are shown below. Abbreviations: P35S, CaMV 35S promoter; G148^R^, NPTII gene conferring G418 resistance; SUL^R^, sulfadiazine resistance gene. (**B**) Phylogenetic tree of ataxia-telangiectasia-mutated (ATM) and ATM and Rad3-related (ATR) proteins of representative lineages including mammals (Hs, *Homo sapiens*; Mm, *Mus musculus*), insects (Dm, *Drosophila melanogaster*), yeasts (Sc, *Saccharomyces cerevisiae*; Sp, *Schizosaccharomyces pombe*), flowering plants (At, *Arabidopsis thaliana*; Os, *Oryza sativa*), and bryophytes (Pp, *Physcomitrella patens*; Spf, *Sphagnum fallax*). Bootstrap values are shown.

The protein sequences deduced from these sequences were compared to the entire collection of non-redundant protein sequences in the European Nucleotide Archive (ENA)/GenBank collection using BLASTP with default search parameter settings (https://blast.ncbi.nlm.nih.gov/Blast.cgi). These searches exclusively retrieved ATM and ATR sequences at the top significance levels with ATM and ATR as query from a large variety of different organisms. All other members of the PIKK family such as PRKDC or mTOR followed with far less significant identity scores, thus confirming the identity of these proteins as ATM and ATR. As an additional confirmation of their identity, the same genes are annotated as Pp*ATM* ( Pp3c2_23700V3.1) and Pp*ATR* (Pp3c6_3460V3.1) in the version v3.3 models [33] available in Phytozome (*Physcomitrella patens* v3.3, DOE-JGI, http://phytozome.jgi.doe.gov/) [34], which are identical to them except for the 5′ and 3′ untranslated regions. In addition, ATM and ATR protein sequences from representative organisms and sequenced plant genomes were retrieved from the National Center for Biotechnology Information (NCBI) and Phytozome databases and the phylogeny of these proteins across representative organisms were established (Figure 1B) using AlignX (VectorNTI suite, Thermo Fisher Scientific) with the default settings of the program.

### 2.2. Production of Ppatm and Ppatr Mutants 

Gene replacement vectors were designed to produce a deletion of 1809 bp in the *PpATM* gene, which removes the last 6 exons (*Physcomitrella patens* v3.3, Chr02:15863420..15865228) from the genome to replace it with a sulfonamide resistance gene [24]. The deletion in the *PpATR* gene covers 1680 bp encoding the last 3 exons (*Physcomitrella patens* v3.3, Chr06:1980747.. 1982426) that are replaced by the *NPTII* gene [24]. The corresponding gene replacement vectors were obtained as follows. The 5′ and 3′ regions of homology were obtained by long template PCR (Roche Expand long template PCR kit) using *P. patens* wild type genomic DNA. For *PpATM*, primer pairs were for 5′ ATMup 1 (TTT CCC GGG TCG ACG GAA CAG)/ATMup 2 (CAC CCA TGG ATG AGG AAC ATC AAG TAC) and 3′ ATMdown 1 (GGG GAG CTC ATA GAG GTG AGG CTA AAA G)/ATMdown 2 (CCC CAC TAG TAC GTG TTT AGA GTT CCC TC). Primers included restriction sites (underlined) for *Xma*CI, *Nco*I, *Sac*I, and *Spe*I, which were used to combine these fragments with the sulfonamide resistance gene located on a *Nco*I/*Sac*I fragment obtained from pUC18/Sul [24] and insert them together into *Xma*I/*Spe*I-cut pBluescript. For *PpATR*, primer pairs were for 5′ ATRup 1 (GTT CTC ATA TCG ATT TGT GCA TAC C)/ATRup 2 (AAA GAA TTC CCC AGT AGA ACA ACC TGA AAC) and 3′ ATRdown 1 (GGT AAG CTT GTG CCA ACG TTT GAT AGT C)/ATRdown 2 (GGG GTC GAC ATT CTC ATT CTT GGG). These primers included restriction sites for *Eco*RI, *Cla*I, *Hind*III, and *Sal*I, which were used to connect these fragments to the *NPTII* gene of pUC18/NPTII [24] located on an *Eco*RI/*Hind*III fragment and insert them into *Cla*I/SalI-digested pBluescript.

To produce the mutants, plasmids were digested with *Xma*I/*Spe*I or *Cla*I/SalI, which liberated the targeting fragments. Transformation into *P. patens* protoplasts and selection of stable transformants were as described [35]. Transformants were characterized in detail by PCR, Southern blotting (Figure S1), and flow cytometry, as described in [24,36]. Gene replacement was detected by 5′ and 3′ recombination border fragment-specific standard PCR (Amplicon Taq polymerase, 40 cycles). The 5′ border fragments were detected with primer pairs PpATM_genome_fw (AAC GCC ATC TGT GCA TTC G)/PpATM/ATR_p35S (AAT TGC CCT TTG GTC TTC TGA G) for ATM and PpATR_genome_fw (TCG GTT GCT TAC CCT TTG G)/PpATM/ATR_p35S for ATR. The 3′ border fragments were detected with primer pairs PpATM_tpSul_fwd/PpATM_genome_re (CAG TTC CAG ACC TTA ACC TTC ATC) for ATM and PpATR_NPTII_fwd/PpATR_genome_re (AAT GAT GTG GCT GTC GCT G) for ATR. To detect transformants with precise replacements, we amplified the entire locus by long template PCR with primer pairs PpATM_genome_fw/PpATM_genome_re for ATM and PpATR_genome_fw/PpATR_genome_re for ATR and modified or unmodified loci identified by the size of the fragments obtained. For Southern blots, genomic DNA was digested with *Sty*I for *PpATM* and *Bso*BI for *PpATR*. Procedures and design (Figure S1) were as described [30]. Probes were obtained by PCR. The primer combinations were for *PpATM*: 5′, sATM_5flank_fwd (GGC TTT AGA ACA CTT GTC CAT)/sATM_5flank_rev (CTC TAG CAC ACC AGC ACT TG); 3′, sATM_3flank_fwd (CTC TTA CTA TCG GAA TGC TCA G)/sATM_3flank_rev (GTG GAG ACG TTA AGC AAG ATG); deleted part, sATM_del_fwd2 (GTT GGA CTT GGA GAT CGA) C)/sATM_del_rev (CAT AAC AGA TAG AGT GGC TTC). For *PpATR*: 5′, sATR_5flank_fwd (TGT CAT CGA GCA ATA GCC GAA C)/sATR_5flank_rev (ACC GAG GCA GGG CTT GAA AC); 3′, sATR_3flank_fwd (CAG CGA CAG CCA CAT CAT T)/sATR_3flank_rev (GCT TAG ATT TAC ATG TGC GAG G); deleted part, sATR_del_fwd (AGG ATG CCC GTA TGA TGG)/sATR_del_re (CTA GTG AGA GCC CTT TGT CG).

Haploid mutants were selected that had correct 5′ and 3′ recombination junctions, the expected deletion, and the precise gene replacement. *Ppatmatr-1* was obtained in a cross of *Ppatm-1* to *Ppatr-1* by selection for both G418 and sulfadiazine resistance, and verification of the cross by PCR.

### 2.3. Plant Growth Assays 

*P. patens* cultures, procedures, and media were described in detail in Zobell and Reiss [35] and correspond to common procedures in the *P. patens* community [21] that we adapted from Schaefer and Zryd [22]. In particular, the two media used here, minimal and standard medium, an ammonium tartrate-supplemented minimal medium, correspond in composition to those described by Schaefer and Zryd [22]. Standard growth conditions were 26 °C with continuous white light from the top in a Percival CU 36L/5D growth cabinet. For growth assays, tissue fragments prepared as for routine sub-culturing were plated at low density on standard media with cellophane overlay. For assays on square plates, 7 day old colonies (referred to as micro-colonies) of comparable size were picked with forceps and transferred in ordered arrays with wild type, *Ppatm-1*, *Ppatr-1*, and *Ppatmatr-1* in 1 row and 5 replicas in the column to 128 x 128 mm square plates containing standard or minimal media. Subsequent growth was under standard conditions and monitoring was in 7-day intervals using a Nikon DS-Fi2-L3 or Leica MZ16 binocular microscope and photography. For direct growth monitoring, freshly prepared tissue fragments were filtered through a 100-micron sieve to enrich fragments consisting of 1-3 living cells, were diluted, and were plated on 9 cm Petri dishes containing standard medium without cellophane overlay. Growth monitoring was as above. Plates were in duplicate for all experiments, and the data shown are based on three independent experiments. 

### 2.4. DNA Damage Resistance and Checkpoint Control Assays 

For the acute assays, protoplasts were isolated as described [35]; were diluted to 20,000 protoplasts per treatment in standard/mannitol media; had bleomycin or mitomycin C added as indicated in the figures; were incubated for 1.5 and 16 hours, respectively; were collected by centrifugation; were washed with standard/mannitol medium; and were processed further as described for protoplast transformation [35]. Regenerating protoplasts were counted as the colonies obtained after 10 days in culture. Survival was calculated relative to the mock-treated sample. For the chronic assays, micro-colonies (wild type, *Ppatm-1*, *Ppatr-1*, *Ppatmatr-1* and *Pprad51AB-1* in one row, 5 replicas per column) were picked on square plates containing bleomycin, mitomycin C, MMS, methylnitrosourea (MNU), and hydroxyurea at the concentrations indicated in the figure and grown for 14 (28 for hydroxyurea) days under standard conditions. Plates were in duplicate and data represent at least two independent experiments. Concentrations are given in units active substance per liter (u/L) for bleomycin, in milligrams per liter (mg/l) for mitomycin C, and in millimolars (mM) for the others. For checkpoint control assays, micro-colonies were transferred to liquid standard medium and incubated for 2 hours in the presence and absence of 3 u/L bleomycin, and then 20 µM 5-ethynyl-2′-deoxyuridine (EDU) was added and culturing continued under constant shaking for an additional 6 hours. Processing was as described in the manufacturer’s instructions (Click-iT EDU Alexa Fluor 488 Imaging Kit, Molecular Probes/Invitrogen). In brief, colonies were fixed, EDU was coupled to Alexa488 Fluor to visualize replicated cells, and nuclei were counter-stained with 4′,6-Diamidine-2′-phenylindole dihydrochloride (DAPI). DAPI counterstained and Alexa488 Fluor-stained tip cells were counted by fluorescence microscopy, and the data were normalized to the DAPI-stained nuclei and plotted.

### 2.5. Compilation of DNA Damage Repair-Related and ATM- and ATR-Interacting Genes from A. thaliana and P. patens 

Genes involved in DNA damage repair were compiled from published datasets described for yeast [37] and human [38], their protein sequences were retrieved from the ENA/GenBank and UniProt databases, and the collection was updated and complemented with ATM- and ATR-interacting genes. The same databases were used to retrieve the corresponding *A. thaliana* orthologues by BLASTP searches using the yeast and human protein sequences as query. This collection was complemented with plant-specific recombination genes retrieved from TAIR (www.arabidopsis.org) and redundancy caused by nomenclature differences between organisms was removed. The collection of 186 different genes obtained this way is presented in Table S1. To identify the corresponding genes in the *P. patens* genome, we BLASTed the entire collection of DNA repair genes comprising all the yeast, human, and *A. thaliana* protein sequences against the version 1.6 gene models, which was available at CosMoss.org at that time, and through using the tools this site provided. The corresponding hits were manually curated and ambiguities were resolved with additional BLAST analyses in the NCBI and UniProt databases using unclear sequences as query. Remaining uncertainties in ontology were clarified by phylogenetic analyses in representative species and unambiguous genes were listed individually, or otherwise they were grouped together. This analysis resulted in a collection of 120 different genes with functions related to DNA repair that are present in *P. patens* (Table S1). These are represented by 188 different sequences due to the gene duplications existing in the *P. patens* genome. A free tool for the conversion of the v1.6 to the v3.3 *P. patens* genome annotation is available at http://plantco.de/research.php.

### 2.6. SuperSAGE and Transcriptomics 

To obtain synchronous, actively growing cultures, we passaged stocks 3 times on standard medium with cellophane overlay by routine sub-culturing with 10 plates, each grown under exactly identical conditions, i.e., in parallel and in the same shelf in the same growth cabinet. Material was pooled, sub-cultured again on 10 plates with standard medium and cellophane overlay for 5 days, harvested, and pooled again. For the analysis of the transcriptional response of wild type to bleomycin, an aliquot (approximately 1 gram wet weight) was treated for 1 hour with 0, 0.3, and 3.0 units/liter (u/L) bleomycin in 10 ml liquid standard medium in a 50 ml Falkon tube before RNA was prepared. An additional aliquot of the 0.3 u/L treatment was washed free of bleomycin by filtering through a nylon mesh, washing with water, and incubated for additional 3 hours in 20 ml standard medium in a 9 cm Petri dish before RNA was prepared immediately thereafter. For the mutant analysis, wild type, *Ppatm-1*, *Ppatr-1*, and *Ppatmatr-1* cultures were prepared as described above and the material pooled. Mock-treatment and treatment with 0.3 u/L bleomycin was with an aliquot (approximately 5 gram) by incubation in 300 ml liquid standard media in a 500 ml Duran bottle followed by removal of bleomycin by filtering and washing and an additional 3 hour incubation in fresh standard medium before RNA was prepared immediately thereafter. Preparation of total RNA was as described [39]. SuperSAGE analysis and data processing were performed by GenXPro (Frankfurt, Germany), as described in [40,41]. In brief, libraries were prepared and were sequenced using Illumina technology, tag counts were normalized to tags per million, the corresponding *P. patens* transcripts were identified by BLAST searches in the version 1.6 release gene models [42], and the corresponding transcript abundance frequencies were calculated. For the whole genome analysis, transcript abundance frequencies were processed with ArrayStar (Lasergene DNASTAR Bioinformatics software suite) using default parameter settings. For the collection of DNA repair genes, we pooled non-identical gene models hit by the same tag sequence in families, aggregated tag counts within the same gene, and excluded ambiguous data and genes represented with less than 10 tags. Finally, transcript abundance frequencies were used to calculate abundance ratios between wild type and mutants, and induced and non-induced conditions.

### 2.7. Gene Targeting Assays 

Gene targeting assays were as described [30]. Wild type, *Ppatm-1*, *Ppatr-1*, and *Ppatmatr-1* protoplasts were transformed in single experiments in parallel. Detection of gene targeting was by PCR, and transformants with at least one correct 5′ or 3′ recombination junction were counted as positive. The data are the mean of 7 independent experiments. Differentiation between gene replacement, one-sided integration, and ectopic targeting was performed by PCR with a representative subset. 

### 2.8. Accession Numbers

Sequence data from this article can be found in the ENA/GenBank data libraries under accession numbers LT999877 (*Pp*ATM, Pp3c2_23700V3.1) and LT999878 (*Pp*ATR, Pp3c6_3460V3.1). The original SuperSage data consisting of tag sequences together with associated tag frequency counts are available at ArrayExpress under accession number E-MTAB-9082.

## 3. Results

### 3.1. The P. patens ATM and ATR Orthologues, Gene Structure, and Production of Mutants

The identification of ATM and ATR is described in detail in the Materials and Methods section and it was essentially confirmed that the gene models in the latest *P. patens* chromosome-scale assembly, Pp3c2_23700V3.1 (PpATM) and Pp3c6_3460V3.1 (*PpATR*) [33], are the correct *P. patens* orthologues. Our analysis also confirmed the described gene structures (Figure 1A), except for the extreme 5′ and 3′ ends of the predicted transcripts. The corresponding transcript sequences are available at ENA/GenBank. 

ATM and ATR proteins are well conserved in the three FAT, PI3K, and FAT-C domains characterizing the PIKK family proteins, but have diverged over larger evolutionary distances. To show their correct affiliation and position within the bryophytes and the general plant kingdom, we selected representative ATM and ATR protein sequences from organisms with functionally characterized genes, except for plants, where *A. thaliana* is the only representative and their phylogenies were established (Figure 1B). This analysis confirmed the identity and phylogenetic position of PpATM and PpATR and confirmed a generally lower degree of conservation of ATM as compared to ATR between organisms.

The PI3K domain is essential for function in mammalian genes [43,44,45]. To obtain non-functional ATM and ATR variants, we deleted the genomic regions encoding PI3K and FAT-C domains by targeted gene replacement and exchanged them for two different resistance genes (Figure 1A). The ATM domain replacement mutant was obtained by constructing a targeting vector (described in detail in the Materials and Methods section), which removed the last six exons from the *PpATM* gene and replaced it with a sulfonamide resistance gene [24]. The *PpATR* targeting vector was constructed to remove the last three exons from *PpATR* and replace it with the G418 resistance-conferring *NPTII* gene [24]. Protoplasts were transformed with the corresponding targeting vectors using standard methods [22], and transformants were selected and characterized. Detailed PCR and Southern blotting analyses described in the Materials and Methods section were used to identify individuals with correct 5′ and 3′ recombination junctions in which the intended genomic segments were deleted and precisely replaced with the marker genes. The results are shown in Figure S1. Three independent domain replacement mutants were selected—*Ppatm-1*, *Ppatm-2*, *Ppatm-3* and *Ppatr-1*, *Ppatr-2*, *Ppatr-3*—which were verified to be haploid by flow cytometry (Figure S2). The *Ppatmatr-1* double mutant was obtained by crossing *Ppatm-1* and *Ppatr-1* and selection of the resulting spores for resistance to both G418 and sulfadiazine. The presence of both mutations in the double mutant was verified by PCR.

### 3.2. ATM and ATR are Important for Vegetative Development

An illustration of the complete *P. patens* life cycle is shown in Figure S3, and detailed background information on this organism is available in several reviews [19,20,21,46]. In brief, *P. patens* has a dominant gametophytic, haploid generation with distinct juvenile and adult phases. The former is characterized by two-dimensional growth of protonema, a tissue consisting of two types of filaments: chloronema and caulonema. Outgrowth from a spore or regeneration of tissue begins with the formation of slower-growing chloroplast-rich chloronemata, followed by a gradual transition to faster-growing caulonemata. Side branch formation generates secondary chloronemata and also the bud initials that develop into gametophores, the leafy shoots of the moss plant, at the beginning of the adult phase. Thus, the later stages are characterized by a mixture of protonemal and gametophore growth, both of which determine colony morphology and circumference.

**Figure 2:**
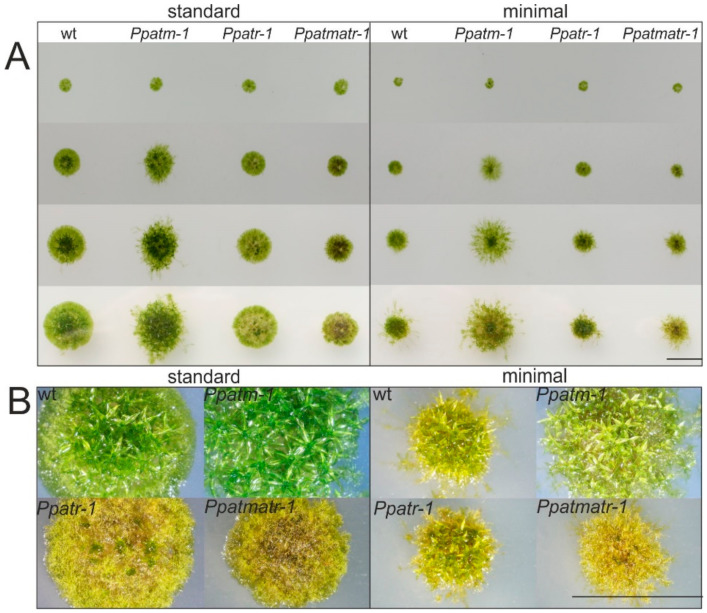
Developmental phenotypes of *Ppatm-1*, *Ppatr-1*, and *Ppatmatr-1* mutants under different growth conditions. (**A**) The pictures show colony growth after transfer of micro-colonies in ordered arrays to standard (left panel) and minimal (right panel) medium under standard conditions. Pictures show colonies after 7 (row 1), 14 (row 2), 21 (row 3), and 28 (row 4) days of growth. (**B**) Magnification of colonies in row 4 showing details in colony morphology and gametophore development. Bars = 1 cm.

To observe vegetative development, 7 day old micro-colonies obtained by sub-culturing of protonema fragments were picked on square plates containing either minimal or standard medium, as well as an ammonium tartrate supplemented minimal medium, and were propagated to the early adult phase. Colony growth of wild-type (wt) and the *Ppatm-1*, *Ppatr-1*, and *Ppatmatr-*1 mutants cultured side-by-side on the same plate was monitored at 7-day intervals by direct observation and photography (Figure 2A). Additionally, tissue fragments were plated directly on media in standard Petri dishes and growth was monitored in a similar manner (Figure S4). *Ppatm-1* colonies on square plates with standard medium differed from wild type in morphology and size from the 14 day time point on. At this stage, caulonemal filaments protruded from the center in the mutant whereas wild type colonies showed more compact central gametophore formation with peripheral protonemata. Regeneration of directly plated tissue fragments showed that caulonemal development was stimulated at the earliest stages of filament regeneration in the *Ppatm-1* mutant. Consequently, the gametophores (Figure 2A) preferentially developing along caulonemata were spread evenly across the surface of the colony in the mutant while their growth was restricted to the center in wild type. *Ppatr-1* mutants initially showed apparently normal growth (Figure 2A), but the chloronema to caulonema transition and branching was noticeably inhibited (Figure S4A). The phenotype became macroscopically visible after the switch from protonema to gametophore development. Gametophore development was severely disrupted, with most bud initials developing into callus-like structures or largely reduced shoot-like structures that gradually degenerated (Figure 2B and Figure S4A). Moreover, filaments rapidly turned brown, a typical feature of ageing [46], which spread throughout the entire colony at later stages. Tissue browning commenced at the beginning of gametophore growth, but is unlikely to be a direct result of growth inhibition [47], suggesting that ATR loss causes this premature ageing phenotype. Thus, ATR became essential for viability following the switch from two- to three-dimensional growth, equivalent with the development of morphologically more complex structures. The phenotype of the *Ppatmatr-1* double mutant closely resembled that of *Ppatr-1*, but was more severe, with growth rate further restricted and gametophore development being almost entirely abolished.

The three independently isolated mutants, *Ppatm-1, -2,* and *-3,* and *Ppatr-1, -2* and *-3* (Figure S4B), showed consistently identical growth phenotypes, indicating that the observed phenotypes resulted from the mutations introduced by the respective domain replacements in the corresponding genes.

Cultures on standard or minimal medium differ in colony morphology and growth rate. Wild type on minimal medium formed smaller colonies but showed enhanced caulonemal development (Figure 2A).This effect was mainly due to the absence of ammonium tartrate in this medium that allows unrestricted caulonema growth and reduces growth rate [19,46]. Considering the altered growth characteristics on this medium, the *Ppatm-*1 phenotype remained unchanged, but increased caulonemal growth rate led to larger colonies and early gametophore development. In the *Ppatr-*1 mutant, the defects in protonemal development were also more pronounced, but principally remained the same. However, later, colonies showed no signs of premature ageing and gametophores did develop (Figure 2B), albeit frequently with aberrant morphology. Since caulonemal development was largely inhibited in *Ppatr-*1, the higher growth rates obtained on standard medium likely caused the browning of colonies and inhibition of gametophore growth. The *Ppatmatr-1* double mutant appeared similar to the *Ppatr-*1 mutant, but the phenotype was more severe and gametophores were virtually absent. The gradual increase in severity of *Ppatr-*1 phenotypes from minimal to standard medium to the *Ppatmatr-*1 double mutant on the same media suggests that the growth-promoting component contributed by the *Ppatm-1* mutant caused the increase in severity of the phenotype. In addition, incubation under light and temperature conditions that reduced growth rate altered or alleviated the observed phenotypes (Figure S5). This suggests that the importance of ATR for viability and development of more complex morphological structures may critically depend on cell proliferation rate, and the data show that growth conditions critically impact the expressivity of mutant phenotypes. 

*Ppatm-1* plants were fully fertile, and gametophores, gametangia, and the sporophyte developed normally (Figure S6), suggesting that ATM plays no role in gametogenesis, fertilization, sporophyte development, and meiosis. Conversely, *Ppatr-1* plants were mostly sterile, with mature gametophores bearing almost no spore capsules, although archegonia and antheridia (the female and male reproductive organs) were produced. However, archegonia were either mostly defective or aborted prematurely, possibly after fertilization had occurred, while antheridia appeared normal (Figure S6) and were clearly able to produce viable spermatozoids, as demonstrated by the productive cross to *Ppatm-1* to obtain the double mutant. Thus the ATR mutation became progressively irreversibly lethal with the onset of sporophyte development throughout reproductive development. The *Ppatmatr-1* mutant was even more defective, and both archegonia and antheridia on the few shoots that developed were morphologically aberrant (Figure S6) with no sporophytes being formed.

### 3.3. ATR Dominates DNA Damage Repair and Checkpoint Control 

**Figure 3:**
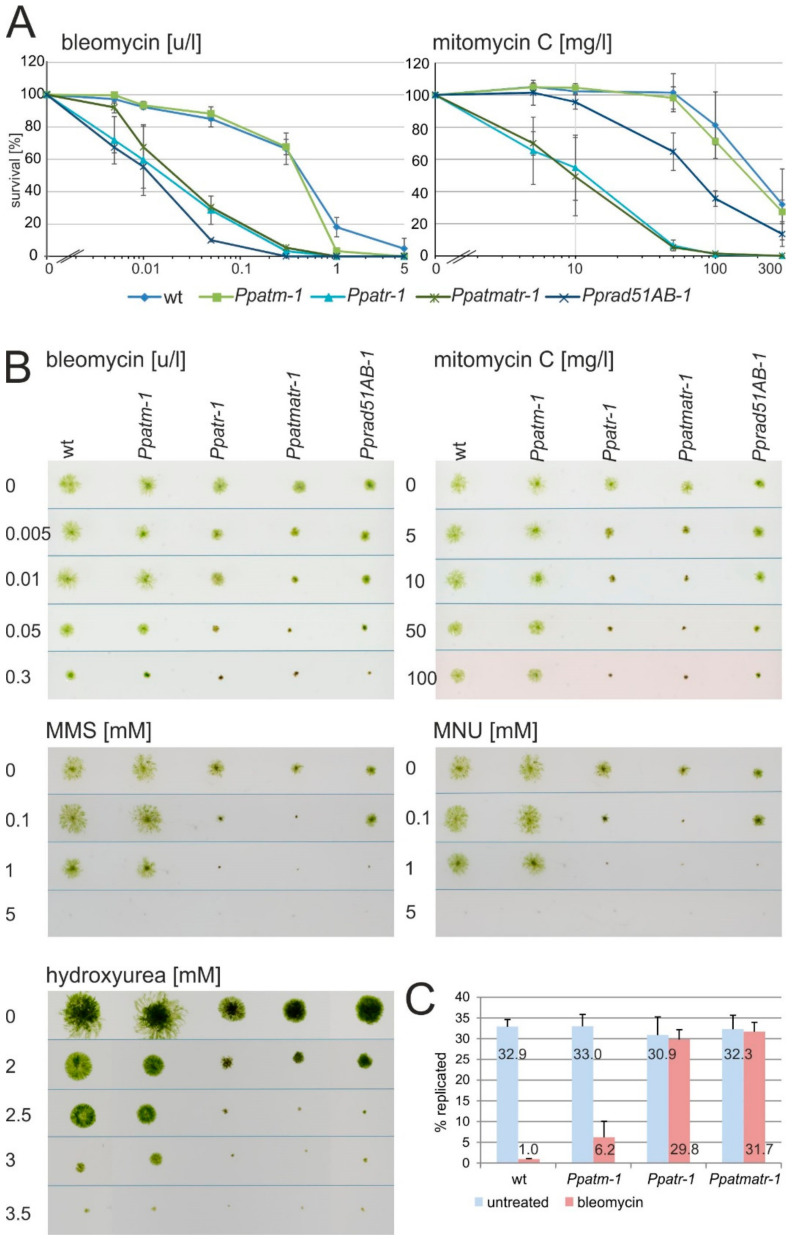
DNA damage resistance and checkpoint control assays. (**A**) Acute assays. The survival curves for wild type and *Ppatm-1*, *Ppatr-1*, *Ppatmatr-1*, and Pp*rad51AB-1* mutants calculated as a percentage relative to the mock treatment are shown in log [10] scale. Data are the mean (*n* = 2) +/- SD. (**B**) Chronic assays. The pictures show colonies after 14 (28 for hydroxyurea) days of growth in the presence of the indicated concentrations of genotoxins under standard conditions. (**C**) Checkpoint control assays. The graph shows the number of replicated (Alexa488-fluorescent) cells as the percentage of the total number of stainable (DAPI-positive) cells in 20 independent colonies in mock (untreated) and bleomycin-treated (bleomycin) plants. Data are the mean (*n* = 3) +/- SD. Concentrations are in units active substance per liter (u/L), milligrams per liter (mg/l), or millimolars (mM).

We used chronic and acute exposure to different genotoxins to analyze the role of ATM and ATR in DNA damage repair. These chemicals induce a spectrum of damages that are specific for each substance, but all of them finally induce DSBs. The radiomimetic bleomycin causes DSBs directly (direct DSBs), whereas the lesions induced by the others (mitomycin C, MMS, MNU, and hydroxyurea) are processed to DSBs during replication (replication-coupled DSBs). In acute assays, protoplasts were pulse-treated with increasing concentrations of bleomycin or mitomycin C, were allowed to recover in the absence of the drugs, and had the survivors scored. This assay (Figure 3A) showed that both wild type and *Ppatm-1* mutants were equally resistant to bleomycin and mitomycin C while both *Ppatr-1* and *Ppatmatr-1* mutants were highly sensitive. Additionally, the survival curve indicated that all DNA repair capacity was lost in these two mutants. Thus, ATM contributed neither to the repair of direct nor replication-coupled DSBs or other DNA damage under these conditions, whereas ATR alone mediated recovery from DNA damage including both types of DSBs. Complete abolition of RAD51 activity in the *Pprad51AB-1* double mutant [24] caused the same degree of sensitivity to bleomycin as *Ppatr-1* and *Ppatmatr-1*, suggesting that repair of direct DSBs is by an HR pathway regulated by ATR. However, the *Pprad51AB-1* double mutant was only partially sensitive to mitomycin C, suggesting that lethality was not exclusively caused by replication-coupled DSBs, but that other forms of DNA damage played a role also or that RAD51-mediated HR plays a limited role in replication-coupled DSB repair in *P. patens*, in contrast to *A. thaliana* [24]. Notwithstanding this difference, the *Ppatr-1* survival curve indicated that all DNA damage repair depended on ATR.

In the chronic assays, micro-colonies were inoculated on square plates containing increasing concentrations of bleomycin, mitomycin C, MMS, MNU, or hydroxyurea, and colony growth and morphology scored as a measure of resistance. This assay (Figure 3B) largely recapitulated the results of the acute assay, except that *Ppatm-*1 mutants grew noticeably more slowly than wild type at the highest concentrations of bleomycin and the *Ppatmatr-1* double mutant also appeared more sensitive than *Ppatr-1*. At these concentrations, *Ppatm-*1 colonies arrested growth with reduced caulonemal production, but remained green and viable, like the wild type. This is a mild but noticeable sensitivity to bleomycin in actively growing tissue, predominantly causing growth arrest but not immediate lethality. By contrast, *Ppatr-1* and *Ppatmatr-1* mutants showed early growth arrest and lethality with increasing bleomycin and mitomycin C concentrations, suggesting that ATR is essential for resisting DNA damage and sustained viability alike. These data suggest that ATM has a minor function in the repair of direct DSBs and other damage that is restricted to actively and continuously proliferating cells. All mutants showed a similar pattern of sensitivity to mitomycin C, hydroxyurea, MMS, and MNU, with only the *Ppatr-1* and *Ppatmatr-1* mutants exhibiting significant hypersensitivity. These chemicals cause either inter-strand crosslinks (mitomycin C) or alkylate DNA (MMS and MNU), or directly interfere with replication (hydroxyurea) and have a common outcome in inducing replication-coupled DSBs, either indirectly by conversion of pre-existing lesions to DSBs or directly by causing replication errors. A problem with these assays is the prolonged incubation time and chemical instability of some chemicals (MNU, bleomycin) that causes decay of activity over time such that the material is not exposed to identical concentrations all the time. Nevertheless, the similarity of the results obtained with these chemicals suggests that this problem has limited influence on the performance of such assays. In addition, the similarity in resistance patterns suggests that the lethality inducing lesions limiting survival under these conditions are predominantly replication-coupled DSBs, although the other DNA damage induced by these chemicals clearly requires repair also. Therefore, identical sensitivity to all four chemicals predominantly reflects the different requirements for ATM and ATR in the repair of replication-coupled DSBs, but does not exclude the participation of ATR in the control of other repair pathways dealing with the DNA damage induced by these chemicals also. 

ATM and ATR have a highly conserved function as cell-cycle checkpoint control kinases. This role was analyzed by monitoring replication in protonemal tip cells immediately following exposure to bleomycin, using EDU incorporation and identification of replicating cells [48] by fluorescence microscopy after a short interval (Figure 3C). In the mock-treated sample, the same number of wild type and mutant cells went through replication in this interval, indicating that none of them had a noticeable inherent cell-cycle defect, consistent with the wild type-like flow cytometric histograms obtained with the mutants (Figure S2). Replication in this interval was 97% inhibited in wild type after exposure to bleomycin, showing successful checkpoint activation and cell-cycle arrest in response to DNA damage. This number dropped to 3% and 2% inhibition for *Ppatr-1* and *Ppatmatr-*1, respectively, and the majority proceeded through replication, indicating a significant loss of checkpoint control. In *Ppatm-*1, 81% of the cells remained arrested in replication, indicating that checkpoint control remained largely intact. Nevertheless, the proportion proceeding through replication was notably higher than in wild type, in agreement with the observed mild bleomycin sensitivity of *Ppatm-*1 in proliferating cells.

### 3.4. The Role of ATM and ATR in Gene Targeting

**Figure 4:**
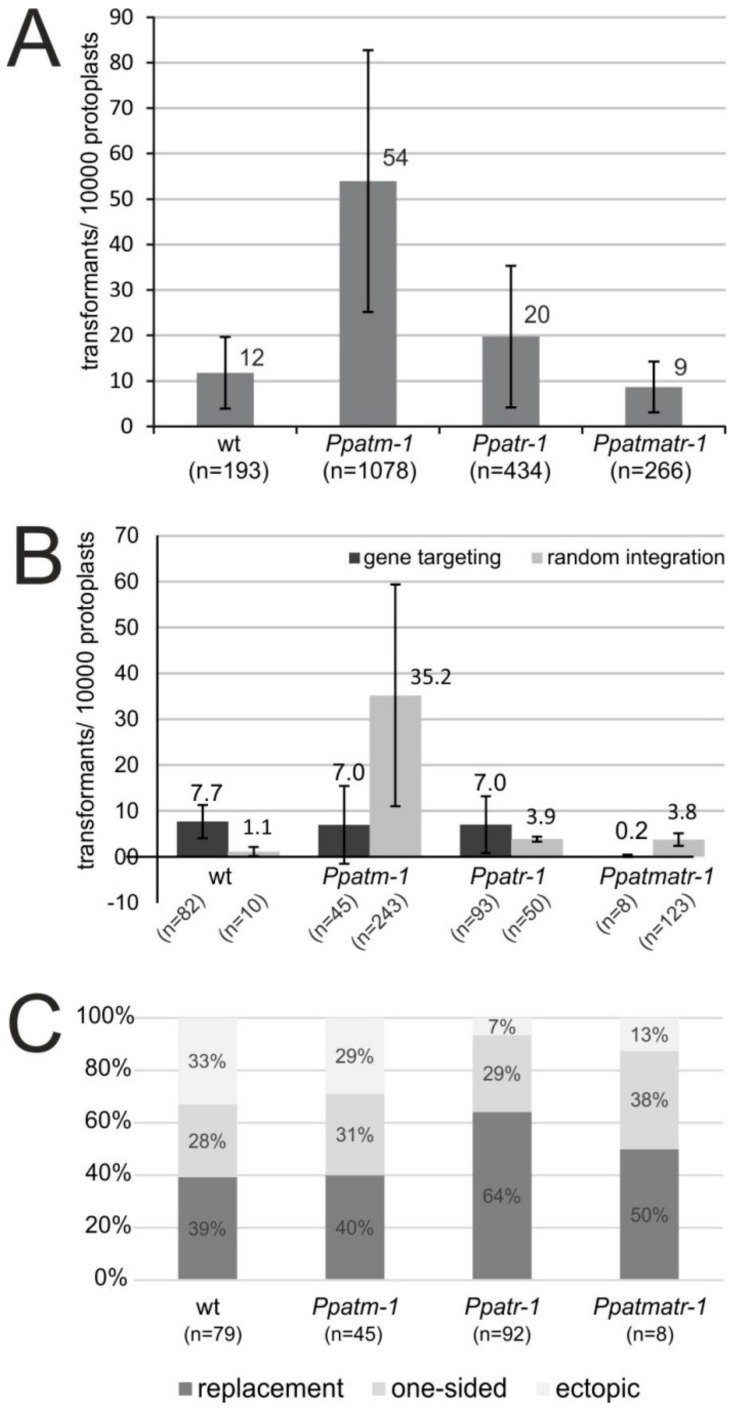
Gene targeting assays. (**A**) The graph shows the number of stable transformants normalized to transformants per 10,000 regenerating protoplasts. The data are the mean of seven independent experiments +/- SD. (**B**) Proportion of gene targeting and random integration events in wild type and the mutants as determined by 5′ and 3′ recombination junction-specific PCR in a representative subset of transformants. The data are the mean +/- SD. (**C**) Relative proportions of gene replacement, one-sided integration, and ectopic targeting in wild type and the mutants, as determined by PCR.

Gene targeting assays were performed with the *PpCOL2* gene as a target, as described previously [30]. Transformation of *Ppatm-*1 yielded significantly more transformants than wild type (Figure 4A) and the absolute number of gene-targeting events per experiment seemed to be reduced to half the number obtained in wild type (Figure 4B). The *PpATR-1* mutation caused a moderate increase in transformation efficiency, but seemed to have no effect on gene targeting. However, gene targeting efficiencies almost dropped below detection levels in the *Ppatmatr-1* double mutant, whereas transformation efficiencies were only mildly affected, if at all. HR-mediated DNA integration processes in *P. patens* produce three different outcomes, gene replacement, one-sided integration, and ectopic targeting (as explained in [30]), together referred to as gene targeting here. An analysis differentiating between these events showed that they also occur in the mutants. The proportions as they occurred in wild type remained the same in *Ppatm-1*, but were shifted from ectopic targeting to gene replacement in *Ppatr-1* and possibly *Ppatmatr-1* also (Figure 4C). This shift indicated an increase in HR-mediated integration of recombination intermediates into the genome and suggests that ATR plays a role in the control of gene targeting after the initiation step. Together, the data suggest that both genes play an unexpected role in the integration of foreign DNA, with ATM being significantly more important than ATR. However, individually both of them have limited importance for gene targeting, with ATM possibly being involved in the initiation step and ATR in the processivity of the process. 

### 3.5. The Importance of ATM and ATR in the Transcriptional Response to Bleomycin-Induced Damage

**Figure 5:**
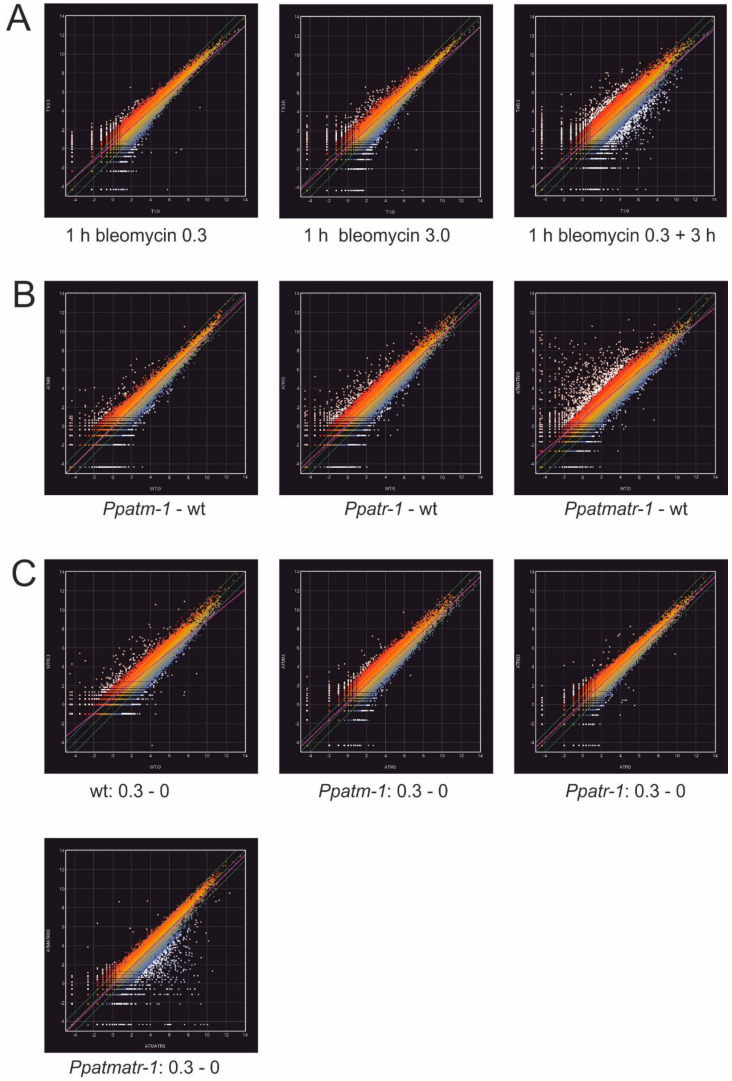
Whole transcriptome SuperSAGE analysis. (**A**) Whole transcriptome analysis of the early response to bleomycin-induced DNA damage. The results of the treatment and mock treatment, respectively, of wild type with lethal (3.0 u/L) and sub-lethal (0.3 u/L) concentrations of bleomycin for 1 hour and the following 3 hour recovery period are shown. The SuperSAGE tags were mapped to *P. patens* transcripts and had their data normalized, and fold change ratios for the 15561 transcripts in the analysis were calculated. The scatter plots show the following comparisons: 1 h bleomycin 0.3, mock treatment for 1 h (T1/0, *x*-axis) versus 1 h 0.3 u/L treatment (T1/0.3, *y*-axis)); 1 h bleomycin 3.0, mock treatment for 1 h (T1/0, *x*-axis) versus 1 h 3.0 u/L treatment (T1/3.0, *y*-axis)); 1 h bleomycin 0.3 + 3 h, mock treatment for 1 h (T1/0, *x*-axis) versus 1 hour 0.3 u/L treatment followed by 3 h recovery (T4/0.3, *y*-axis). (**B**,**C**) Whole transcriptome analysis of mutants in the absence and presence of bleomycin-induced DNA damage. Wild type and *Ppatm-1*, *Ppatr-1*, and *Ppatmatr-1* mutants were treated and mock-treated with a sub-lethal (0.3 u/L) concentration of bleomycin for 1 h followed by a 3 h recovery period, and fold change ratios were calculated from normalized SuperSAGE tags mapped to *P. patens* transcripts as above. (**B**) The scatter plots show mutant to wild type comparison in the absence of bleomycin-induced DNA damage. *Ppatm-1* – wild-type (wt): wt mock-treated (wt0, *x*-axis) versus *Ppatm-1* mock-treated (ATM0, *y*-axis), *Ppatr-1*– wt: wt mock-treated (wt0, *x*-axis) versus *Ppatr-1* mock-treated (ATR0, *y*-axis), *Ppatmatr-1* – wt: wt mock-treated (wt0, *x*-axis) versus *Ppatmatr-1* mock-treated (ATMATR0, *y*-axis). (**C**) The scatter plots show the comparisons of mock- to bleomycin-induced DNA damage for wild type and mutants. wt: 0.3 – 0: wt mock-treated (wt0, *x*-axis) versus treated with 0.3 u/L bleomycin (wt0.3, *y*-axis), *Ppatm-1*: 0.3 – 0: mutant mock-treated (ATM0, *x*-axis) versus treated with 0.3 u/L bleomycin (ATM0.3, *y*-axis), *Ppatr-1*: 0.3 – 0: mutant mock-treated (ATR0, *x*-axis) versus treated with 0.3 u/L bleomycin (ATR0.3, *y*-axis), *Ppatmatr-*1: 0.3 – 0: mutant mock-treated (ATMATR0, *x*-axis) versus mutant treated with 0.3 u/L bleomycin (ATMATR0.3, *y*-axis). Axes are in log2 scale. The purple broken line is the linear regression. The border limits of twofold changes are marked with green lines. Data points with fourfold or more differences between conditions are marked in white. Top and bottom data points are in red and blue, respectively.

To analyze the early phase of the transcriptional response to bleomycin-induced damage and its dependence on the severity of the damage, we used exposure for 1 hour to a sub-lethal (0.3 u/L) and a lethal (3 u/L) dose of bleomycin. In addition, an aliquot of the 0.3 u/L treatment was washed free of bleomycin and incubated for an additional 3 hours in its absence. Thereafter total RNA was prepared and transcriptional patterns were determined by SuperSAGE [49,50]. The original SuperSAGE data comprising tag sequences and associated tag frequencies are available in ArrayExpress. Mapping of tag sequences to *P. patens* v1.6 transcripts [42] showed that a 1 hour treatment with a sub-lethal concentration was sufficient to induce massive changes in expression (Figure 5A). Exposure to the lethal dose provoked an apparently rather similar response. A significantly larger number of genes appeared to be differentially expressed after the 3 hour period following the sub-lethal dose in the absence of bleomycin, but the mock treatment used as reference here was the untreated 1 hour control experiment used for all treatments in this series. Therefore, and as suggested by a comparison with the experiment using identical reference points below (Figure 5C) the treatment procedure alone likely contributed to the increase in differential expression. Consequently, the data in this experiment comprise transcripts of genes responsive to bleomycin-induced damage, which changed in expression within the recovery period and those of stress-responsive genes that react to the treatment.

To analyze the involvement of ATM and ATR in the transcriptional response to DNA damage, we chose the 1 hour bleomycin treatment followed by the 3 hour recovery period. For this analysis, wild type and the *Ppatm-1*, *Ppatr-1*, and *Ppatmatr-1* mutants were cultivated under identical conditions; material from independent plates were pooled, pulse-treated, or mock-treated for 1 hour with 0.3 u/L bleomycin; and total RNA was prepared after the 3 hour recovery period that followed. The SuperSAGE analysis was as described above and the original data are available in ArrayExpress also. The expression profiles (Figure 5B) obtained in the absence of bleomycin-induced DNA damage differed between wild type and the mutants and between them. In this comparison, the double mutant was the one exhibiting most of the changes, displaying a substantial fraction of upregulated transcripts, suggesting that ATM and ATR synergistically control transcript homeostasis of a large number of genes, a substantial fraction of which may comprise treatment stress-responsive genes differentially expressed in the mutants.

A substantial number of transcripts responded to the DNA damage induced by bleomycin in wild type (Figure 5C). This response is noticeably affected in the mutants and fewer transcripts responded in the *Ppatm-1* and *Ppatr-1* mutants, or expression changes were lower. The *Ppatmatr-1* double mutant stood out by showing a markedly different expression pattern, which was almost a mirror image of the pattern found in the absence of DNA damage before (Figure 5B). The noticeable differences between the single and the double mutants suggest that the response to bleomycin-induced DNA damage is synergistically coordinated by ATM and ATR also. However, the changes in expression between different conditions and the mutants and wild type by far exceeded the number of known DNA repair genes, suggesting that many of them reflect secondary effects not directly related to DNA damage repair or general stress responses.

To focus on the analysis of DNA repair functions and ATM and ATR interacting proteins, we collected the genes involved in these processes in yeast (*Saccharomyces cerevisiae*) [37] and human [38] and retrieved the corresponding orthologues in *P. patens* and *A. thaliana*, as described in the Materials and Methods section. This collection is shown in Table S1. The transcript abundance ratios between wild type and mutants and induced and non-induced conditions were calculated from the SuperSAGE data for the genes in this set and are presented in Table 1. The corresponding data for the different bleomycin induction conditions in wild type are shown in Table S2. All mutants showed differences in their expression profiles to wild type in the absence of bleomycin-induced DNA damage (Table 1). However, only a few genes were altered in expression in *Ppatm-1*, while the changes in *Ppatr-1* and *Ppatmatr-1* were more substantial. In addition, the *Ppatmatr-1* profile shows only few similarities to either single mutant.

Bleomycin treatment induced massive alterations in the expression profiles in this set. The most interesting genes induced in wild type were early responding DNA damage repair activities and components of the two major DSB repair pathways, HR and NHEJ (Table 1 and Table S2). One of the early induced activities was the 9-1-1 complex. This complex is involved in early steps of DSB repair; consists of hRAD1, HUS1, and RAD9A; and plays a prominent role in damage recognition and in recruitment of repair proteins to the damaged site [51,52]. All of these genes, including the *RAD17* gene whose activity loads the complex onto DNA, were induced, albeit to different levels. PARP family proteins were also among the activities early responding to DNA damage, having DNA break-sensing and signaling activities involved in multiple repair pathways [53]. One of them, *PARP2*, the gene functionally important in *A. thaliana* [54], was also noticeably induced. Of the two main DSB repair pathways, the key players in HR—*RAD51*, *RAD54*, and *RAD54B*—and in classical NHEJ—*KU70* and *KU80*—were induced. In addition, many if not all other DNA repair pathways were also induced (Table 1 and Table S2). Among these were genes with general functions such as processing of “dirty” DSBs (*PNKP*) and sealing of a DSB (*XRCC4*), or activities mediating interaction with chromatin (*SMC6*) and activation of cell-cycle checkpoints (*MDC1*). These are largely the same principal activities that were described as being induced by a 24 hour bleomycin treatment previously [55].

The response in the *Ppatm-1* mutant was rather similar to wild type (Table 1). In particular, genes encoding components of the 9-1-1 complex (*HUS1*, *RAD9A*), *PARP2*, and the two main DSB repair pathways remained inducible, although their expression levels changed. In addition, expression levels of other genes also changed (Table 1), showing that ATM is relevant, but likely plays only a minor role in the control of direct DSB repair. By contrast, upregulation of the early responding activities and DSB repair pathways was noticeably more defective in the *Ppatr-1* mutant, and additionally expression of many more genes was affected (Table 1). The *Ppatmatr-1* mutant was noticeably different from any of the single mutants. Induction of almost all genes failed, and expression levels above the un-induced state were mostly elevated basal levels. This pattern confirms the complexity of the roles of ATM and ATR in the DDR and stresses their tight interaction and synergistic relationship in the transcriptional regulation of the DDR.

## 4. Discussion

The mutations in the *P. patens ATM* and *ATR* genes were produced by deletion of the PI3K and FAT-C domains located in the carboxyl-terminal regions of the proteins. The PI3K kinase domain is essential for function in mammalian cells [43,44,45], suggesting that the mutated proteins are unable to phosphorylate any downstream target proteins, their only well-documented activity. Consequently, *Ppatm-1* and *Ppatr-1* are likely loss of function mutants, but both genes may still be expressed and the remaining protein portions retain a residual function. Such mutants may differ in phenotype from mutants created in different ways, as generally observed with different mutations in the same gene.

Both *Ppatm-1* and *Ppatr-1* displayed phenotypes during their vegetative growth phase (Figure 2). The *Ppatm-1* mutant progressed slightly faster through the natural succession of growth transitions, but was otherwise normal. The *Ppatr-1* mutant displayed a severe and complex phenotype with all developmental stages affected, beginning with the chloronema to caulonema transition. Later, and and in dependence on growth conditions, the establishment of morphologically complex structures such as gametophores and sporophytes was progressively inhibited in *Ppatr-1* and even more so in *Ppatmatr-1*, but was normal in *Ppatm-1*. These growth aberrations may have originated in the defects in the DDR (Table 1) and/or cell-cycle checkpoint control (Figure 3C) that are more severe in *Ppatr-1* and *Ppatmatr-1* and could affect long-term viability. Sporadic cell death in complex structures like the gametophore, gametangia, and the diploid sporophyte could substantially cause growth aberrations and consequently cause the phenotypes observed in these mutants. By contrast, mutations in *ATM* or *ATR* in *A. thaliana* cause no obvious vegetative phenotype, in line with mutations in other recombination genes (e.g., *RAD51*) [24,25,26,27,28,29] that cause phenotypes in *P. patens* but not in *A. thaliana*. The differences in ATM and ATR and the other recombination genes suggest a tight connection of DNA damage repair to developmental processes in *P. patens* that does not exist in *A. thaliana*, independently of the mechanisms that may cause these phenotypes. The role of ATM in meiosis also seems to differ between *P. patens* and *A. thaliana*, in line with the differences in somatic recombination. ATM is important in the recombinational processing of programmed meiotic DSBs [13] in *A. thaliana*, but not in *P. patens*, as suggested by the unaltered sporulation capacity of the *Ppatm-1* mutant. 

An intriguing feature of *P. patens* is the high efficiency of gene targeting. Our analysis showed that neither gene alone was critical for gene targeting, but together they were. Although *ATM* is not important for gene targeting in mammalian cells also [56], this is a surprising result for *ATR*, given its leading role in the DDR. A variety of recombination activities [27,28,29], including those with a central role in HR, RAD51, and RAD51 paralogue B, [24,25,26,30], have been analyzed in *P. patens*. Collectively, these analyses suggest that gene targeting and direct DSB repair use common recombination pathways in *P. patens*, at least up to the point where HR diverges [6,7] into the gene conversion and crossover HR sub-pathways. The concurrent dependence of resistance to bleomycin and upregulation of main DSB repair activities (9-1-1, PARP2, HR, and NHEJ) on ATR suggests that transcriptional regulation plays an essential role in direct DSB repair. Gene targeting efficiency is barely affected in the *Ppatr-1* mutant, but almost completely lost in the *Ppatmatr-1* double mutant. However, the main DSB repair activities are not significantly more affected than in *Ppatr-1* (Table 1), suggesting that transcriptional regulation of DSB repair pathways plays no or only a minor role in gene targeting. This may be so because sufficient amounts of repair activities pre-exist to support gene targeting, but not DSB repair. Alternatively, ATM and ATR may redundantly be involved in transcriptional control of other repair pathways or mechanisms that are less important for DSB repair but critical for gene targeting, such as the switch between gene conversion and crossover HR. However, the repertoire of ATM and ATR functions is not limited to transcriptional regulation. Phosphorylation of downstream targets is an important function to consider as well as the involvement of these kinases in other stress responses and cell-cycle control. Consequently, any of these could be relevant for gene targeting and/or could be the primary determinant of the unexpected and yet unexplained increase in transformation efficiency in both single mutants (Figure 4A). Whatever these functions in detail may be, our data point to important differences in the regulatory circuits controlling direct DSB repair and gene targeting and consequently in the functions of ATM and ATR between them.

ATM and ATR are important in cell-cycle checkpoint control in mammals and *A. thaliana* [15]. In *P. patens*, ATM has limited importance in checkpoint control and ATR is dominant. ATR also dominates transcriptional regulation in the DDR of *P. patens* and controls induction of early responding components (9-1-1 complex, PARP2) and the major DSB repair pathways (HR and NHEJ) (Table 1). Moreover, ATR almost exclusively controls the establishment of resistance to bleomycin while ATM’s contribution seems to be restricted to proliferating cells. The minor role of ATM is consistent with the minimal importance of NBS1, a direct ATM interaction partner involved in the recognition of direct DSBs in DNA damage repair in *P. patens* [27]. The relative roles of ATM and ATR are comparable to their counterparts in yeast. Tel1 (ATM) contributes only marginally to direct DSB repair, has little importance in checkpoint control [57], and plays only a secondary role in signaling pathways [58,59], whereas these processes are dominated by Mec1 (ATR). This suggests that the *P. patens* proteins have yeast-like functions with ATR as the master coordinator in the DDR. By contrast, *A. thaliana atm* mutants display marked sensitivity to direct DSB-inducing agents and the transcriptional response is largely dependent on ATM [13,14,15,17,18], indicating that ATM and ATR are functionally specialized like in mammals. These differences imply a substantial divergence of ATM and ATR function between *A. thaliana* and *P. patens*. However, although similar, *P. patens* ATM and ATR functions are not identical to yeast since signaling in metazoa and yeast is largely mediated through two important acceptor kinases, CHK1 and CHK2 (RAD53) [2,8,10,60], both of which do not exist in the *A. thaliana* and *P. patens* genomes (Table S1). Their absence suggests major differences in the DDR between plants and other eukaryotes, indicating additional differences in ATM and ATR downstream signaling and consequently in their function in cell-cycle checkpoint control and DNA damage repair. The absence of the third PIKK in the DDR, PRKDC, in *A. thaliana* and the BRCA2 and BRCA1 orthologues in *P. patens* suggests that such differences also exist between plants (Table S1). These differences suggest a high degree of divergence in important components of the DDR and downstream DNA damage repair pathways across major eukaryotic lineages and, consequently, the existence of major mechanistic differences among them. 

*A. thaliana* and *P. patens* share a common ancestor, but have different preferences for direct DSB repair pathways—NHEJ in *A. thaliana* and mammals and HR in *P. patens* [24] and yeast—with the high efficiency of gene targeting possibly reflecting the latter preference. The correspondence of DSB repair pathway choice to yeast- or human-like ATM and ATR features in these organisms suggests that these are the adaptations in the DDR necessary to follow these preferences. *P. patens* is a relatively direct descendant of the first genetically haploid, land-colonizing plants. The conditions prevailing at that time likely caused massive intracellular genotoxic and general stress, for instance through the production of reactive oxygen species or by constant dehydration/rehydration cycles. These conditions may have required special mechanisms for the maintenance of genome stability that were maintained by selection through the environmental conditions in its present day habitat. HR for direct DSB repair together with cell-cycle arrest in G2 or late S phase [36,61] provides a mechanism for precise and efficient DNA damage repair. A homologous DNA template that is obviously lacking in the haploid vegetative phase of *P patens* is provided by shortening of the G1 phase such that cell-cycle arrest preferentially occurs in G2 or late S phase, thereby providing a sister chromatid for homology-mediated DSB repair most of the time. Inhospitable conditions faced by yeast in its natural environment [62] may have similarly promoted the use of HR for DSB repair and consequently the adaptation of ATM and ATR to this preference. These connections suggest that yeast-like ATM and ATR features could have relevance for gene targeting.

## 5. Conclusions

In essence, the divergence in ATM and ATR features between *A. thaliana* and *P. patens*, as well as other organisms, are likely adjustments in the DDR required for the alternate use of DSB repair pathways that is driven by differing requirements for the maintenance of genome stability under different environmental conditions. Such an evolutionary selection mechanism predicts a much higher diversity in ATM and ATR functions between species that is entirely independent of their affiliation to individual taxonomic lineages. In support of this hypothesis, human-like genes exist in all vertebrates and *A. thaliana* and exist in yeast-like forms in yeast, fission yeast (*Schizosaccharomyces pombe*), the insect *Drosophila melanogaster*, and the bryophyte *P. patens* [3,10,13,16,60,63], suggesting that such diversity indeed exists. Moreover, their existence in disparate eukaryotic lineages such as metazoa, plants, and yeast suggests that these forms have evolved independently multiple times in several different lineages and kingdoms.

## Figures and Tables

**Table 1:** DNA damage repair gene-specific differential expression profiles.

	1		2			3			4	
Gene (UniProt nomenclature)	wt	*Ppatm-1*			*Ppatr-1*			*Ppatmatr-1*		
	wt Bleo/wt0	atm0/wt0	atm Bleo/wt0	atm Bleo/atm0	atr0/wt0	atr Bleo/wt0	atr Bleo/atr0	atmatr0/wt0	atmatr Bleo/wt0	atmatr Bleo/atmatr0
Checkpoints, Regulators, and Signaling										
ATRIP	0.8	0.9	0.5	0.6	0.8	0.8	1.0	1.0	0.9	0.8
ATRX	1.4	1.3	3.0	2.3	1.2	1.4	1.1	2.2	2.2	1.0
hRAD1/REC1	0.6	0.6	0.4	0.7	0.4	0.6	1.6	0.4	0.4	0.9
Hus1	2.6	0.7	3.8	5.1	1.8	1.2	0.7	2.0	1.8	0.9
MAPK family	1.5	0.9	0.6	0.7	1.0	1.2	1.1	1.2	1.6	1.3
MDC1	2.6	0.6	1.9	3.0	2.0	1.6	0.8	3.8	2.1	0.6
PARP2	6.0	0.8	2.6	3.5	1.0	1.2	1.2	3.0	0.7	0.3
PMYT1	0.6	1.0	0.9	0.8	1.0	1.0	1.1	1.2	0.9	0.8
RAD17	2.2	1.4	1.5	1.1	1.7	1.7	1.0	1.6	1.7	1.1
RAD9A/Sprad9	21.0	0.4	10.4	27.6	2.6	2.4	0.9	4.7	2.3	0.5
WEE1	0.9	0.7	0.9	1.4	1.1	1.7	1.5	0.7	1.1	1.7
Homologous Recombination and Meiosis										
HOP2	0.9	0.8	0.8	1.0	0.7	0.9	1.2	0.9	0.9	1.1
MUS81	0.3	0.7	0.2	0.3	0.3	0.2	0.6	0.8	0.6	0.8
NBN	0.8	1.0	0.7	0.6	0.7	0.7	1.0	0.7	0.6	0.9
PaxIP1 (1)	0.8	0.8	0.7	0.8	1.0	0.8	0.9	1.8	0.8	0.4
PaxIP1 (2)	3.1	1.6	0.7	0.4	0.5	2.4	4.5	3.0	0.5	0.2
PaxIP1 (3)	0.5	0.7	0.7	1.0	0.9	0.6	0.6	1.1	1.0	0.9
RA51B	0.4	1.0	1.0	1.0	1.4	1.5	1.0	0.9	1.4	1.6
RA51C	1.8	0.7	0.8	1.0	1.3	1.6	1.2	1.2	1.3	1.1
RA54B	2.8	0.7	2.9	4.2	0.8	1.5	1.9	1.2	1.1	0.9
RAD50	0.7	1.6	1.5	1.0	0.7	1.2	1.6	1.3	1.0	0.8
PpRAD51A	5.2	0.9	5.4	6.0	0.8	2.1	2.7	1.5	1.7	1.1
PpRAD51B	1.0	0.5	1.9	3.8	1.7	1.2	0.7	1.7	1.2	0.7
RAD54 (1)	2.8	0.7	3.5	4.9	1.4	1.6	1.2	2.1	2.2	1.0
RAD54 (2)	0.5	1.3	0.8	0.6	0.5	0.7	1.4	1.5	1.5	1.0
RMI2	1.4	0.9	2.2	2.4	1.4	1.6	1.2	1.4	1.8	1.3
RQSIM	1.4	0.8	1.4	1.8	0.9	1.8	2.0	1.5	1.5	1.0
SPO11	2.2	1.5	2.6	1.8	2.9	2.0	0.7	1.9	3.5	1.8
TOP3B	0.2	0.6	0.8	1.3	1.0	1.3	1.4	0.5	1.0	1.8
WRIP1 (1)	2.1	0.6	1.7	3.0	2.3	0.7	0.3	1.9	1.7	0.9
WRIP1 (2)	0.5	0.3	0.3	1.1	0.6	0.7	1.3	0.7	0.5	0.7
WRX (1)	1.3	1.1	1.5	1.4	3.3	2.1	0.6	3.0	1.7	0.6
WRX (2)	1.2	1.1	1.6	1.5	2.2	1.0	0.4	1.9	1.2	0.6
XRCC2	0.7	0.6	0.9	1.6	0.7	0.9	1.2	1.6	1.5	1.0
General Functions and Crosslink Repair										
DNL1	1.5	1.0	0.9	0.8	0.6	0.8	1.4	0.8	0.8	0.9
FANCL	1.0	1.0	0.5	0.5	0.7	0.5	0.8	0.8	0.8	1.0
PIF1	2.8	1.3	1.5	1.1	1.9	1.1	0.6	2.4	2.6	1.1
PNKP	4.6	0.5	5.5	10.6	0.4	1.3	3.3	2.7	2.9	1.1
RAD21	0.5	0.7	1.1	1.5	0.5	0.8	1.5	0.7	0.5	0.7
RNF4 (1)	1.0	0.7	0.7	0.9	0.4	0.4	0.9	0.7	0.7	0.9
RNF4 (2)	1.1	1.4	1.5	1.1	1.3	1.1	0.8	2.9	1.6	0.5
RNF4 (3)	0.8	0.8	0.7	0.8	0.6	0.7	1.1	0.9	1.1	1.2
RUVBL1	0.4	1.6	1.4	0.9	0.9	0.4	0.5	1.3	1.2	0.9
RUVBL2	0.4	1.0	0.6	0.6	0.7	0.9	1.4	0.6	0.9	1.6
SCC2	0.6	1.4	1.1	0.8	0.7	0.9	1.3	1.0	0.8	0.8
SMC1A	1.3	0.9	1.6	1.8	2.1	1.4	0.6	1.5	1.2	0.8
SMC3	1.1	1.0	1.2	1.3	1.7	1.6	0.9	1.3	0.9	0.6
SMC5	0.6	0.7	0.6	0.9	0.7	0.9	1.2	0.7	0.7	0.9
SMC6	3.5	0.4	3.9	8.8	1.3	0.8	0.6	1.7	0.4	0.2
STAG1	1.3	0.5	1.3	2.5	0.4	1.0	2.4	1.2	0.6	0.5
Non-Homologous End-Joining										
KU70/XRCC6	7.8	0.8	3.6	4.5	0.9	2.3	2.6	2.8	1.1	0.4
KU80/XRCC5	5.3	0.9	4.3	4.8	2.5	2.3	0.9	3.8	3.3	0.9
PRKDC	0.9	1.3	1.3	1.0	0.9	0.9	1.1	0.5	0.4	0.8
XRCC1	0.8	1.0	0.8	0.8	0.8	0.6	0.7	1.4	0.4	0.3
XRCC4	5.1	0.2	2.5	11.1	0.3	1.2	4.8	0.4	0.7	1.6
Mismatch Repair										
EME1	0.6	1.3	1.6	1.2	2.3	3.0	1.3	1.4	3.0	2.1
EXO1	1.2	1.0	1.3	1.4	1.4	1.6	1.2	1.3	1.5	1.2
MLH1	1.7	0.8	1.2	1.4	1.0	1.2	1.1	1.4	0.9	0.7
MLH3	2.5	0.8	1.0	1.2	0.8	1.2	1.6	1.4	1.0	0.7
MSH2	1.3	0.4	1.5	3.9	1.4	1.3	1.0	1.8	1.5	0.9
MSH3	1.4	1.0	1.2	1.2	1.3	0.9	0.7	1.5	0.7	0.5
MSH5	0.9	0.9	1.1	1.3	1.1	1.5	1.4	1.3	1.3	1.0
MSH6	0.4	0.4	0.9	2.2	0.4	1.1	2.5	0.9	0.7	0.8
Muts	0.6	1.5	1.5	1.0	1.5	1.3	0.9	2.2	2.3	1.1
PMS1/PMS2	0.7	1.0	0.7	0.7	0.5	0.8	1.6	0.7	0.7	1.1
Nucleotide Excision, Base Excision, and UV Repair										
DDB1 (1)	1.1	1.0	0.9	0.9	0.8	0.7	0.8	0.8	1.0	1.3
DDB1 (2)	0.8	1.0	1.5	1.5	1.4	1.2	0.9	1.1	1.1	1.0
DDB2	3.6	0.9	2.5	2.8	1.6	1.5	0.9	2.9	1.0	0.3
ERCC1/Rad10 (1)	0.8	0.9	1.0	1.1	0.8	0.9	1.1	1.2	1.1	0.9
ERCC1/Rad10 (2)	1.1	0.3	0.4	1.3	1.3	0.2	0.2	5.0	0.3	0.1
ERCC2/XPD	2.4	0.5	1.8	3.6	1.1	0.9	0.8	1.6	1.8	1.1
ERCC3/Rad25/XPB	1.0	0.7	1.2	1.7	1.0	0.9	0.9	0.8	1.1	1.5
ERCC5/RAD2	1.0	1.1	1.0	0.9	1.0	1.1	1.1	0.9	1.0	1.1
ERCC6/rad26 (1)	4.8	1.3	4.5	3.5	2.3	2.7	1.2	3.1	1.8	0.6
ERCC6/rad26 (2)	0.8	0.8	0.8	0.9	0.6	0.9	1.5	0.9	0.9	1.0
RAD16 (1)	1.3	0.7	2.2	3.1	1.8	1.8	1.0	2.2	1.9	0.9
RAD16 (2)	0.6	1.4	1.9	1.3	1.5	1.3	0.8	1.5	1.5	1.0
RAD23 (1)	0.5	0.9	0.7	0.7	0.7	0.8	1.3	0.8	0.8	1.0
RAD23 (2)	0.4	1.2	1.1	0.9	1.1	0.8	0.7	1.0	1.5	1.5
RAD23 (3)	1.9	1.1	1.2	1.1	1.6	0.7	0.4	2.4	1.1	0.5
RAD27/FEN1 (1)	3.5	1.3	1.7	1.3	0.9	2.0	2.3	2.0	2.8	1.4
RAD27/FEN1 (2)	2.1	0.8	1.4	1.7	0.4	1.5	4.3	1.4	1.0	0.7
RAD6/UBC2 (1)	1.0	1.1	1.1	1.1	1.1	1.1	1.0	1.5	1.1	0.7
RAD6/UBC2 (2)	1.0	0.6	0.7	1.1	0.8	0.9	1.1	0.8	0.8	1.0
SYF1	1.5	1.1	0.8	0.7	1.1	1.4	1.3	1.3	1.6	1.2
XPC/Rad4	1.1	0.3	2.9	10.0	1.5	1.6	1.1	0.2	0.8	4.2
XPF/ERCC4/RAD1	1.5	0.5	1.4	3.0	1.2	1.1	1.0	1.1	1.4	1.3
ATM and ATR Interactors										
Cdc5l	1.4	0.6	1.6	2.9	2.0	1.4	0.7	1.1	1.8	1.6
CHD family (1)	0.5	0.8	1.1	1.3	1.0	0.7	0.8	1.0	0.8	0.8
CHD family (2)	0.5	1.0	0.8	0.8	0.8	1.0	1.3	0.9	1.0	1.1
E2F1	1.0	0.8	0.6	0.7	1.0	0.7	0.8	1.2	1.2	0.9
H2A(1)	1.1	1.4	2.0	1.4	1.4	1.1	0.8	1.5	1.4	0.9
H2A (2)	0.8	1.0	1.4	1.4	0.8	0.8	1.0	0.9	1.0	1.0
HDAC1 (1)	0.5	0.6	1.3	2.2	0.7	0.9	1.3	0.8	0.8	1.0
HDAC1 (2)	1.3	1.1	0.9	0.8	0.9	1.0	1.1	1.3	1.8	1.3
HDAC1 (3)	1.2	0.8	1.4	1.9	1.3	1.1	0.8	2.7	1.2	0.5
MCM7	0.5	1.4	0.5	0.3	0.7	0.6	0.9	0.5	1.1	2.5
PTPA (1)	1.0	0.7	1.1	1.7	0.7	0.8	1.0	0.9	1.0	1.0
PTPA (2)	0.1	0.5	0.4	0.7	0.3	0.1	0.5	0.7	0.6	0.8
RENT1 (UPF1) (1)	0.4	0.6	1.0	1.7	0.8	1.5	1.9	0.6	1.5	2.6
RENT1 (UPF1) (2)	1.1	1.1	0.7	0.6	1.1	1.0	0.9	0.3	1.5	4.2
RFA1 (RPA)	1.2	0.8	2.3	2.8	0.8	1.1	1.4	1.1	0.8	0.8
RFA2 (RPA)	1.1	1.3	0.8	0.6	1.4	1.4	1.0	1.2	1.4	1.2
SOSB1/hSSB1	0.6	1.2	1.1	0.9	0.8	1.4	1.6	0.6	0.8	1.3
STRAP (1)	0.6	1.1	0.4	0.4	1.3	0.7	0.6	0.9	0.7	0.8
STRAP (2)	0.7	0.7	0.7	0.9	0.8	0.8	1.1	0.9	0.7	0.8
TERF family (1)	0.6	0.7	0.4	0.6	0.5	0.6	1.2	0.5	0.6	1.2
TERF family (2)	1.8	1.1	1.9	1.7	1.9	1.4	0.7	1.9	0.8	0.4
TERF family (3)	0.8	0.9	0.6	0.6	1.0	0.9	0.9	1.0	1.1	1.1
TERF family (4)	2.2	0.8	1.4	1.7	1.9	2.5	1.3	2.1	2.2	1.1
TERF family (5)	1.9	0.7	0.9	1.3	1.0	1.0	1.0	1.3	0.3	0.2

Column 1 shows the wild type differential expression profile obtained after bleomycin induction expressed as the transcript ratio (fold change) between bleomycin-treated and -untreated samples (wtBleo/wt0). The corresponding data for the mutants are shown in columns 2, 3, and 4. Basal transcript profiles in the absence of DNA damage (fold change mutant/wt0) follow differential expression profiles calculated for the changes relative to untreated wild type (fold change mutant Bleo/wt0) and untreated mutant (fold change mutant Bleo/mutant (0). Twofold and higher changes in expression are highlighted, with increases in red and decreases in blue. Non-identical transcripts of identical orthologues are distinguished by numbers in brackets. Genes are grouped in pathways and names are abbreviated in UniProt nomenclature with human names given preference over yeast, fission yeast, and plants. Full gene names, aliases, and descriptions are available at https://www.uniprot.org/ and https://www.genecards.org/.

## Supplementary Materials

The following are available online at https://www.mdpi.com/2073-4425/11/7/752/s1. Figure S1: Molecular analysis of *Ppatm* and *Ppatr* mutants. Figure S2: Flow cytometric analysis of selected mutants. Figure S3: The *P. patens* life cycle. Figure S4: Supplemental growth analyses. Figure S5: Environmental conditions shape the *Ppatm-1*, *Ppatr-1*, and *Ppatmatr-1* phenotypes. Figure S6: The mature gametophyte and reproductive structures. Table S1: Conservation of DNA damage repair-related genes between human, yeast, and *A. thaliana* and the corresponding gene set in *P. patens*. Table S2: The early response of DNA damage repair-related genes to bleomycin-induced DNA damage.

## References

[1] Tubbs A., Nussenzweig A. (2017). Endogenous DNA Damage as a Source of Genomic Instability in Cancer. Cell.

[2] Melo J., Toczyski D. (2002). A unified view of the DNA-damage checkpoint. Curr. Opin. Cell Biol..

[3] Harper J.W., Elledge S.J. (2007). The DNA Damage Response: Ten Years After. Mol. Cell.

[4] Ciccia A., Elledge S.J. (2010). The DNA Damage Response: Making It Safe to Play with Knives. Mol. Cell.

[5] Hustedt N., Durocher D. (2017). The control of DNA repair by the cell cycle. Nat. Cell Biol..

[6] Heyer W.D., Ehmsen K.T., Liu J. (2010). Regulation of homologous recombination in eukaryotes. Annu. Rev. Genet..

[7] Ceccaldi R., Rondinelli B., D’Andrea A.D. (2016). Repair Pathway Choices and Consequences at the Double-Strand Break. Trends Cell Biol..

[8] Shiloh Y. (2003). ATM and related protein kinases: Safeguarding genome integrity. Nat. Rev. Cancer.

[9] Bensimon A., Aebersold R., Shiloh Y. (2011). Beyond ATM: The protein kinase landscape of the DNA damage response. FEBS Lett..

[10] Blackford A.N., Jackson S.P. (2017). ATM, ATR, and DNA-PK: The Trinity at the Heart of the DNA Damage Response. Mol. Cell.

[11] Cimprich K.A., Cortez D. (2008). ATR: An essential regulator of genome integrity. Nat. Rev. Mol. Cell Biol..

[12] Saldivar J.C., Cortez D., Cimprich K.A. (2017). The essential kinase ATR: Ensuring faithful duplication of a challenging genome. Nat. Rev. Mol. Cell Biol..

[13] Garcia V., Bruchet H., Carnescasse D., Granier F., Bouchez D., Tissier A. (2002). *AtATM* Is Essential for Meiosis and the Somatic Response to DNA Damage in Plants. Plant Cell.

[14] Liu C.H., Finke A., Diaz M., Rozhon W., Poppenberger B., Baubec T., Pecinka A. (2015). Repair of DNA Damage Induced by the Cytidine Analog Zebularine Requires ATR and ATM in *Arabidopsis*. Plant Cell.

[15] Culligan K.M., Robertson C.E., Foreman J., Doerner P., Britt A.B. (2006). ATR and ATM play both distinct and additive roles in response to ionizing radiation. Plant J..

[16] Culligan K., Tissier A., Britt A. (2004). ATR regulates a G2-phase cell-cycle checkpoint in *Arabidopsis thaliana*. Plant Cell.

[17] Hu Z., Cools T., De Veylder L. (2016). Mechanisms Used by Plants to Cope with DNA Damage. Annu. Rev. Plant Biol..

[18] Ricaud L., Proux C., Renou J.-P., Pichon O., Fochesato S., Ortet P., Montané M.-H. (2007). ATM-Mediated Transcriptional and Developmental Responses to γ-rays in *Arabidopsis*. PLoS ONE.

[19] Cove D. (2005). The Moss Physcomitrella patens. Annu. Rev. Genet..

[20] Reski R., Cove D.J. (2004). Physcomitrella patens. Curr. Biol..

[21] Rensing S.A., Goffinet B., Meyberg R., Wu S.-Z., Bezanilla M. (2020). The Moss *Physcomitrium* (*Physcomitrella*) *patens*: A Model Organism for Non-Seed Plants. Plant Cell.

[22] Schaefer D.G., Zryd J.P. (1997). Efficient gene targeting in the moss *Physcomitrella patens*. Plant J..

[23] Goffova I., Vagnerova R., Peska V., Franek M., Havlova K., Hola M., Zachova D., Fojtova M., Cuming A., Kamisugi Y. (2019). Roles of RAD51 and RTEL1 in telomere and rDNA stability in *Physcomitrella patens*. Plant J..

[24] Markmann-Mulisch U., Wendeler E., Zobell O., Schween G., Steinbiss H.H., Reiss B. (2007). Differential requirements for RAD51 in *Physcomitrella patens* and *Arabidopsis thaliana* development and DNA damage repair. Plant Cell.

[25] Schaefer D.G., Delacote F., Charlot F., Vrielynck N., Guyon-Debast A., Le Guin S., Neuhaus J.M., Doutriaux M.P., Nogue F. (2010). RAD51 loss of function abolishes gene targeting and de-represses illegitimate integration in the moss *Physcomitrella patens*. DNA Repair.

[26] Charlot F., Chelysheva L., Kamisugi Y., Vrielynck N., Guyon A., Epert A., Le Guin S., Schaefer D.G., Cuming A.C., Grelon M. (2014). RAD51B plays an essential role during somatic and meiotic recombination in *Physcomitrella*. Nucleic Acids Res..

[27] Kamisugi Y., Schaefer D.G., Kozak J., Charlot F., Vrielynck N., Hola M., Angelis K.J., Cuming A.C., Nogue F. (2012). MRE11 and RAD50, but not NBS1, are essential for gene targeting in the moss *Physcomitrella patens*. Nucleic Acids Res..

[28] Wiedemann G., van Gessel N., Kochl F., Hunn L., Schulze K., Maloukh L., Nogue F., Decker E.L., Hartung F., Reski R. (2018). RecQ Helicases Function in Development, DNA Repair, and Gene Targeting in *Physcomitrella patens*. Plant Cell.

[29] Trouiller B., Schaefer D.G., Charlot F., Nogue F. (2006). MSH2 is essential for the preservation of genome integrity and prevents homeologous recombination in the moss *Physcomitrella patens*. Nucleic Acids Res..

[30] Wendeler E., Zobell O., Chrost B., Reiss B. (2015). Recombination products suggest the frequent occurrence of aberrant gene replacement in the moss *Physcomitrella patens*. Plant J..

[31] Collonnier C., Epert A., Mara K., Maclot F., Guyon-Debast A., Charlot F., White C., Schaefer D.G., Nogue F. (2017). CRISPR-Cas9-mediated efficient directed mutagenesis and RAD51-dependent and RAD51-independent gene targeting in the moss *Physcomitrella patens*. Plant Biotech. J..

[32] Rensing S.A., Lang D., Zimmer A.D., Terry A., Salamov A., Shapiro H., Nishiyama T., Perroud P.-F., Lindquist E.A., Kamisugi Y. (2008). The *Physcomitrella* Genome Reveals Evolutionary Insights into the Conquest of Land by Plants. Science.

[33] Lang D., Ullrich K.K., Murat F., Fuchs J., Jenkins J., Haas F.B., Piednoel M., Gundlach H., Van Bel M., Meyberg R. (2018). The *Physcomitrella patens* chromosome-scale assembly reveals moss genome structure and evolution. Plant J..

[34] Nordberg H., Cantor M., Dusheyko S., Hua S., Poliakov A., Shabalov I., Smirnova T., Grigoriev I.V., Dubchak I. (2014). The genome portal of the Department of Energy Joint Genome Institute: 2014 updates. Nucleic Acids Res..

[35] Zobell O., Reiss B., Meksem K., Kahl G. (2010). Gene targeting as a precise tool for plant mutagenesis. The Handbook of Plant Mutation Screening (Mining of Natural and Induced Alleles).

[36] Schween G., Gorr G., Hohe A., Reski R. (2003). Unique tissue-specific cell cycle in *Physcomitrella*. Plant Biol..

[37] Prakash S., Sung P., Prakash L. (1993). DNA Repair Genes and Proteins of *Saccharomyces Cerevisiae*. Annu. Rev. Genet..

[38] Wood R.D., Mitchell M., Lindahl T. (2005). Human DNA repair genes, 2005. Mutat. Res./Fundam. Mol. Mech. Mutagenes.

[39] Markmann-Mulisch U., Hadi M.Z., Koepchen K., Alonso J.C., Russo V.E.A., Schell J., Reiss B. (2002). The organization of *Physcomitrella patens* RAD51 genes is unique among eukaryotic organisms. Proc. Natl. Acad. Sci. USA.

[40] Barth M.B., Buchwalder K., Kawahara A.Y., Zhou X., Liu S., Krezdorn N., Rotter B., Horres R., Hundsdoerfer A.K. (2018). Functional characterization of the *Hyles euphorbiae* hawkmoth transcriptome reveals strong expression of phorbol ester detoxification and seasonal cold hardiness genes. Front. Zool..

[41] Stuckas H., Mende M.B., Hundsdoerfer A.K. (2014). Response to cold acclimation in diapause pupae of *Hyles euphorbiae* (Lepidoptera: Sphingidae): Candidate biomarker identification using proteomics. Insect Mol. Biol..

[42] Zimmer A.D., Lang D., Buchta K., Rombauts S., Nishiyama T., Hasebe M., Van de Peer Y., Rensing S.A., Reski R. (2013). Reannotation and extended community resources for the genome of the non-seed plant *Physcomitrella patens* provide insights into the evolution of plant gene structures and functions. BMC Genom..

[43] Cliby W.A., Roberts C.J., Cimprich K.A., Stringer C.M., Lamb J.R., Schreiber S.L., Friend S.H. (1998). Overexpression of a kinase-inactive ATR protein causes sensitivity to DNA-damaging agents and defects in cell cycle checkpoints. EMBO J..

[44] Daniel J.A., Pellegrini M., Lee B.-S., Guo Z., Filsuf D., Belkina N.V., You Z., Paull T.T., Sleckman B.P., Feigenbaum L. (2012). Loss of ATM kinase activity leads to embryonic lethality in mice. J. Cell Biol..

[45] Wright J.A., Keegan K.S., Herendeen D.R., Bentley N.J., Carr A.M., Hoekstra M.F., Concannon P. (1998). Protein kinase mutants of human ATR increase sensitivity to UV and ionizing radiation and abrogate cell cycle checkpoint control. Proc. Natl. Acad. Sci. USA.

[46] Reski R. (1998). Development, genetics and molecular biology of mosses. Bot. Acta.

[47] Moody L.A., Kelly S., Rabbinowitsch E., Langdale J.A. (2018). Genetic Regulation of the 2D to 3D Growth Transition in the Moss *Physcomitrella patens*. Curr. Biol..

[48] Kotogany E., Dudits D., Horvath G., Ayaydin F. (2010). A rapid and robust assay for detection of S-phase cell cycle progression in plant cells and tissues by using ethynyl deoxyuridine. Plant Methods.

[49] Matsumura H., Yoshida K., Luo S., Kimura E., Fujibe T., Albertyn Z., Barrero R.A., Krüger D.H., Kahl G., Schroth G.P. (2010). High-Throughput SuperSAGE for Digital Gene Expression Analysis of Multiple Samples Using Next Generation Sequencing. PLoS ONE.

[50] Molina C., Rotter B., Horres R., Udupa S.M., Besser B., Bellarmino L., Baum M., Matsumura H., Terauchi R., Kahl G. (2008). SuperSAGE: The drought stress-responsive transcriptome of chickpea roots. BMC Genom..

[51] Eichinger C.S., Jentsch S. (2011). 9-1-1: PCNA’s specialized cousin. Trends Biochem. Sci..

[52] Parrilla-Castellar E.R., Arlander S.J.H., Karnitz L. (2004). Dial 9–1–1 for DNA damage: The Rad9–Hus1–Rad1 (9–1–1) clamp complex. DNA Repair.

[53] Huber A., Bai P., Murcia J.M.D., Murcia G.D. (2004). PARP-1, PARP-2 and ATM in the DNA damage response: Functional synergy in mouse development. DNA Repair.

[54] Song J., Keppler B.D., Wise R.R., Bent A.F. (2015). PARP2 Is the Predominant Poly(ADP-Ribose) Polymerase in *Arabidopsis* DNA Damage and Immune Responses. PLoS Genet..

[55] Kamisugi Y., Whitaker J.W., Cuming A.C. (2016). The Transcriptional Response to DNA-Double-Strand Breaks in *Physcomitrella patens*. PLoS ONE.

[56] Kass E.M., Helgadottir H.R., Chen C.C., Barbera M., Wang R., Westermark U.K., Ludwig T., Moynahan M.E., Jasin M. (2013). Double-strand break repair by homologous recombination in primary mouse somatic cells requires BRCA1 but not the ATM kinase. Proc. Natl. Acad. Sci. USA.

[57] Mantiero D., Clerici M., Lucchini G., Longhese M.P. (2007). Dual role for *Saccharomyces cerevisiae* Tel1 in the checkpoint response to double-strand breaks. EMBO Rep..

[58] Craven R.J., Greenwell P.W., Dominska M., Petes T.D. (2002). Regulation of Genome Stability by *TEL1* and *MEC1*, Yeast Homologs of the Mammalian *ATM* and *ATR* Genes. Genetics.

[59] Jaehnig E.J., Kuo D., Hombauer H., Ideker T.G., Kolodner R.D. (2013). Checkpoint Kinases Regulate a Global Network of Transcription Factors in Response to DNA Damage. Cell Rep..

[60] Sekelsky J. (2017). DNA Repair in *Drosophila*: Mutagens, Models, and Missing Genes. Genetics.

[61] Ishikawa M., Murata T., Sato Y., Nishiyama T., Hiwatashi Y., Imai A., Kimura M., Sugimoto N., Akita A., Oguri Y. (2011). *Physcomitrella* Cyclin-Dependent Kinase A Links Cell Cycle Reactivation to Other Cellular Changes during Reprogramming of Leaf Cells. Plant Cell.

[62] Liti G. (2015). The fascinating and secret wild life of the budding yeast *S. cerevisiae*. eLife.

[63] Bentley N.J., Holtzman D.A., Flaggs G., Keegan K.S., DeMaggio A., Ford J.C., Hoekstra M., Carr A.M. (1996). The *Schizosaccharomyces pombe rad3* checkpoint gene. EMBO J..

