# Supplementary material for: The Importance of ATM and ATR in Physcomitrella patens DNA Damage Repair, Development, and Gene Targeting
Source: Genes (Basel). 2020 Jul 6;11(7):752. doi: 10.3390/genes11070752 (PMC7397299; doi:10.3390/genes11070752)
Supplement: Supplementary file 1 [file genes-11-00752-s001.pdf]

Supplemental materials

The importance of ATM and ATR in *Physcomitrella patens* DNA damage repair, development, and gene targeting

Martin Martens, Ralf Horres, Edelgard Wendeler, and Bernd Reiss

Supplemental figures

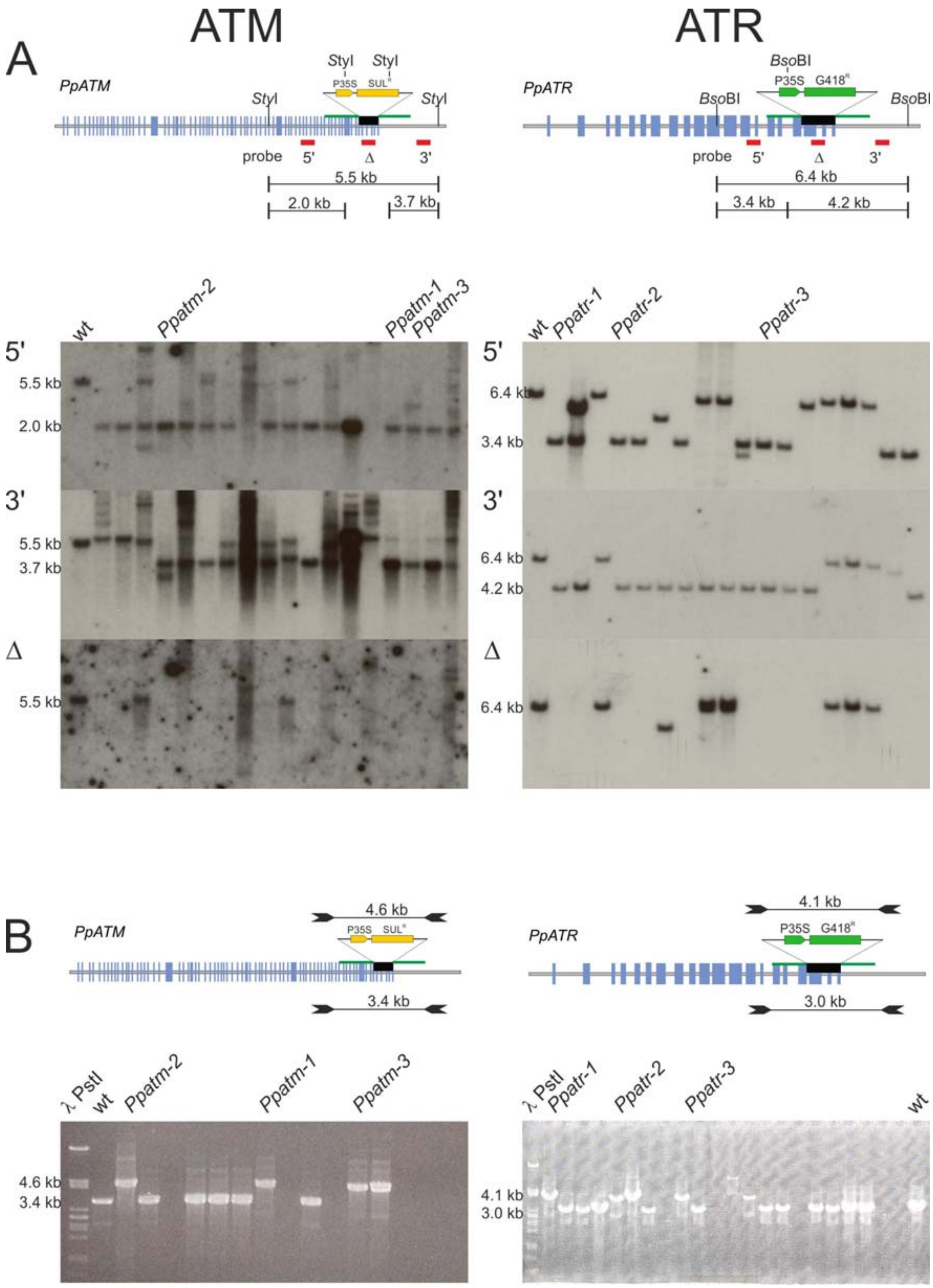

Figure S1: Molecular analysis of *Ppatm* and *Ppatr* mutants. **(A)** Southern blot analysis of mutants. The scheme on top shows show the molecular structure of the *PpATM* and *PpATR* genes

and the gene replacements produced by precise HR, as in figure 1 A. In addition, the regions of homology between vectors and genomic sequences are indicated (green bars). The G418 and SUL resistance marker genes are as in figure 1 A. The positions of restriction enzymes used for the Southern blot analysis are shown. Fragment sizes obtained with unmodified genomic loci and after precise gene replacement are shown below. The results are shown below. Genomic DNA digested with *Sty*I and *Bso*BI, respectively, was separated by agarose gel electrophoresis and blotted to a Nylon membrane. This membrane was probed consecutively with the three different probes detecting the 5’ and 3’ recombination junction fragments and the deleted region (Δ). The blots show that both recombination junctions are correct and the genomic region intended to delete is absent in the selected mutants. **(B)** PCR analysis of mutants. The scheme on top is the same as in A except that positions of primer binding sites and fragment sizes obtained by PCR are indicated. The results are shown below. Genomic DNA was amplified with long template PCR and the primer pairs described in materials and methods, PCR products separated by agarose gel electrophoresis and DNA stained with EtBr. The results show the absence of the unmodified locus and its precise replacement with vectors sequences in the selected mutants. λPst: *Pst*I digested phage Lambda DNA size standard.

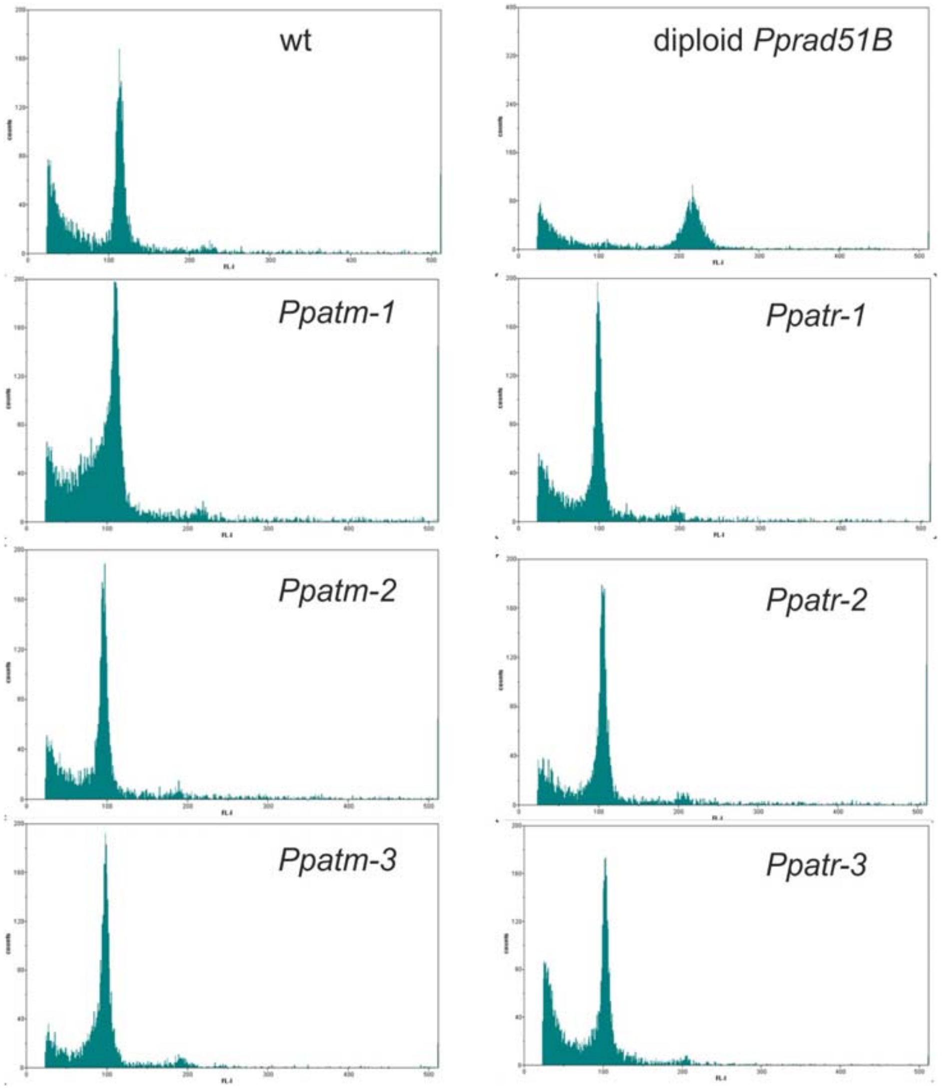

Figure S2: Flow cytometric analysis of selected mutants. Crude nuclear extracts were prepared from 7 day old protonema filaments, nuclei stained with DAPI and sorted by DNA content using flow cytometry as described by the manufacturer (Partek). The *Ppatm-1*, *Ppatm-2*, *Ppatm-3* and *Ppatr-1*, *Ppatr-2*, *Ppatr*-3 flow histograms are identical to wild type while a diploid *Pprad51B* mutant (Markmann-Mulisch et.al. 2007) displays a significantly different profile that lacks the 1C peak entirely. These results confirm that the analysed mutants are haploid.

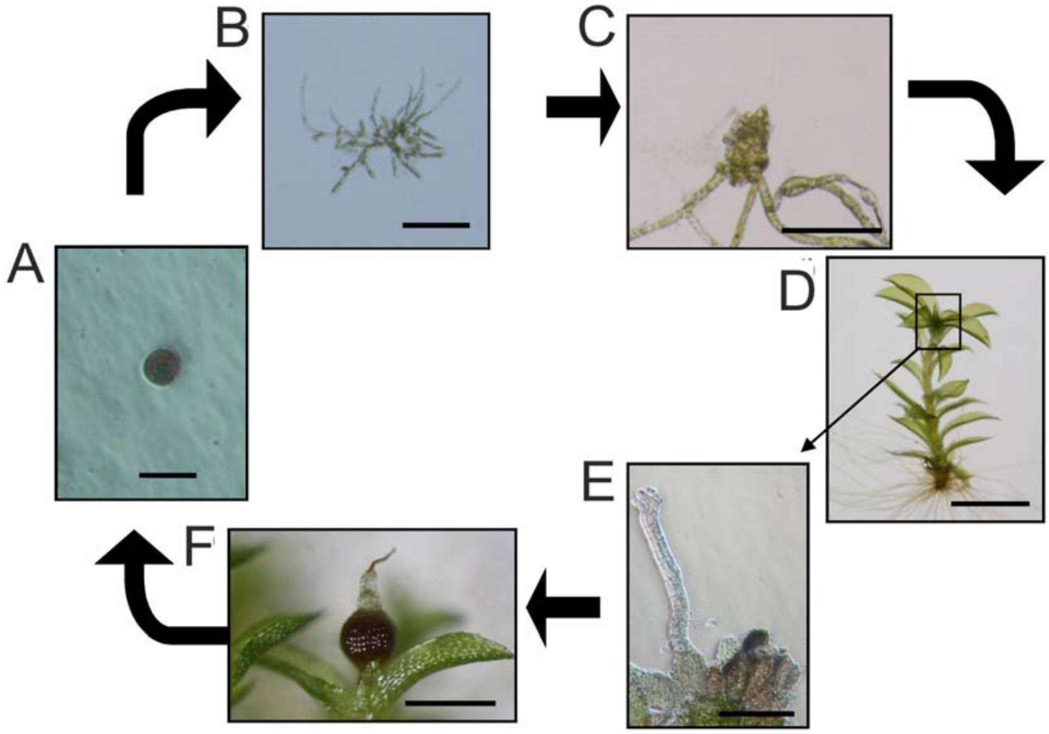

Figure S3: The *P. patens* life cycle. (A) Spore. (B) Protonema filaments consisting of slow growing, chloroplast-rich chloronemata and fast growing chloroplast-poor caulonemata. (C) Buds beginning to develop on caulonema. (D) Mature gametophore with developing (E) Male (antheridium) and female (archegonium) reproductive organs. (F) Mature spore capsule. Scale bars indicate, 10 µm (a), 500 µm (b), 200 µm (c), 1 mm (d), 50 µm (e), 500 µm.

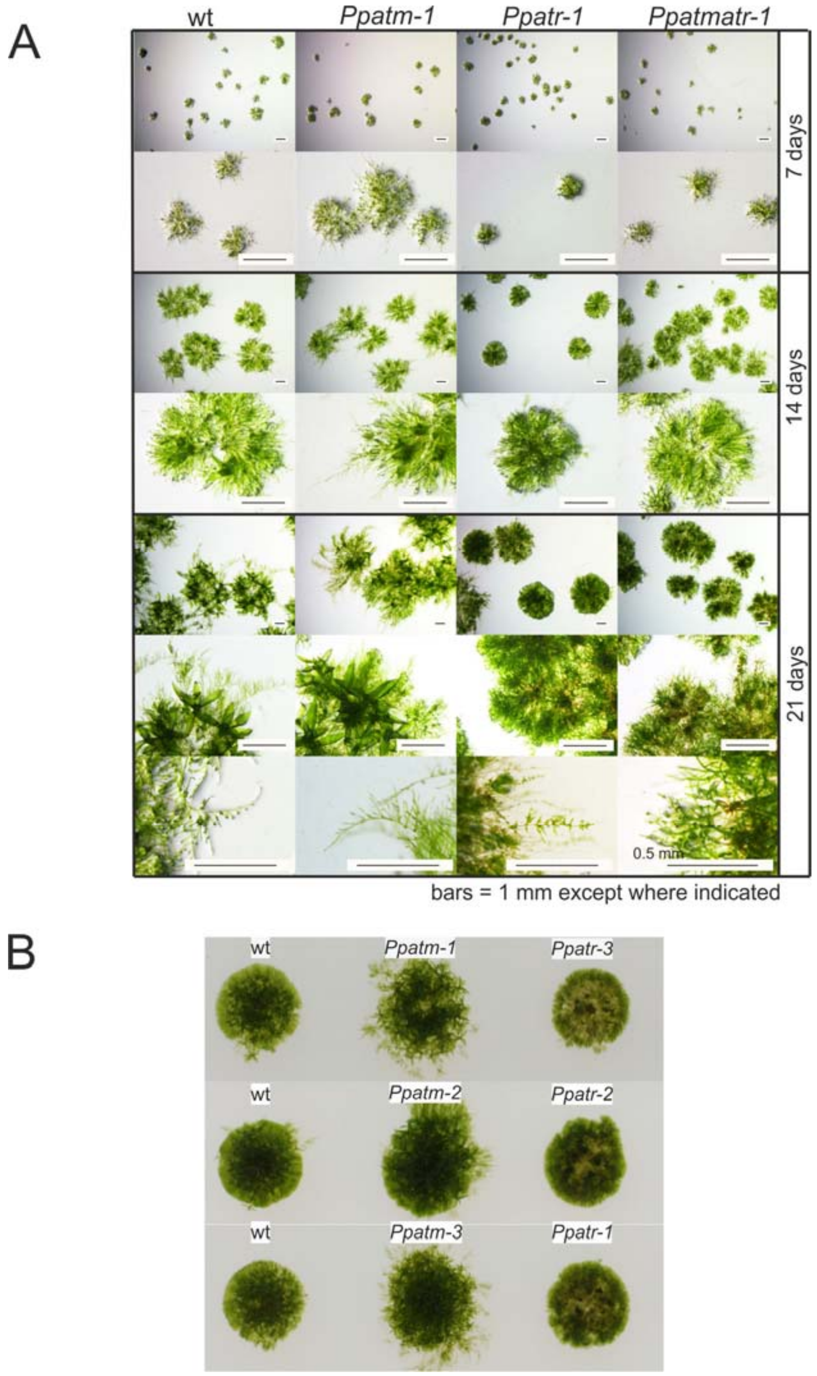

Figure S4: Supplemental growth analyses (A) Early colony growth and protonema phenotypes. Size-selected filament fragments containing 1 to 3 living cells were plated on standard media, cultured under standard conditions and growth monitored. The pictures show colonies after 7, 14 and 21 days. The top rows show overviews and the rows below individual colonies at higher magnification to show details of protonema filament morphology. **(B)** Independently generated *Ppatm-1*, *Ppatm-2*, *Ppatm-3* and *Ppatr-1*, *Ppatr-2*, *Ppatr*-3 mutants display identical phenotypes. The picture shows colonies obtained from micro-colonies on standard medium by culturing under standard conditions for 28 days. The characteristics of the *Ppatm-1* and *Ppatr*-1 phenotypes are identical with their independently generated sisters.

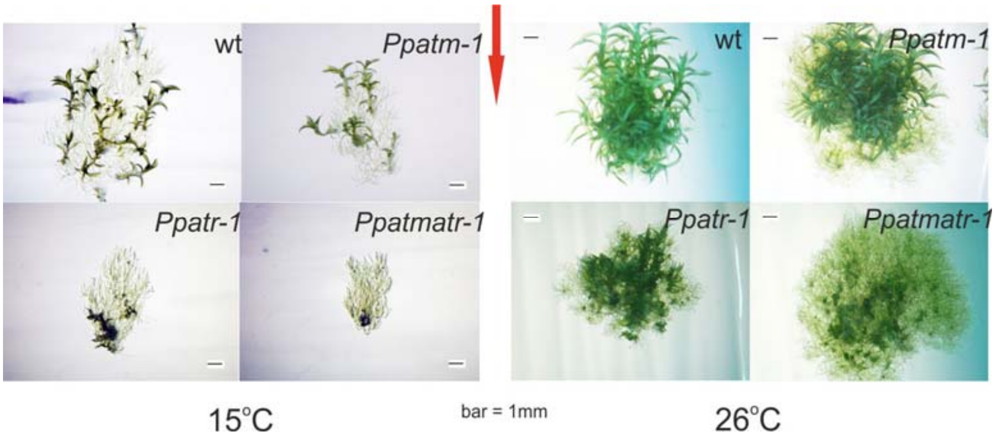

Figure S5: Environmental conditions shape the *Ppatm-1*, *Ppatr-1* and *Ppatmatr-1* phenotypes. Micro-colonies were cultivated on standard media with cellophane overlay in unilateral red light at 15^0C in a 8/16 hour day night cycle or 26^0C in constant light. The pictures show representative colonies after 10 and 5 weeks of growth, respectively. The direction of the red light is indicated by a red arrow.

A

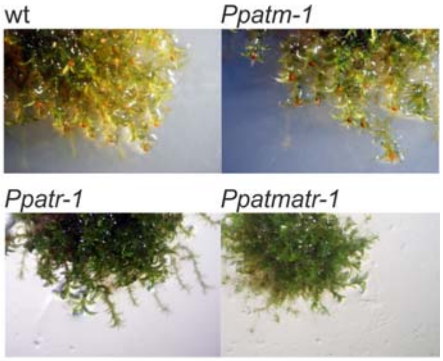

B

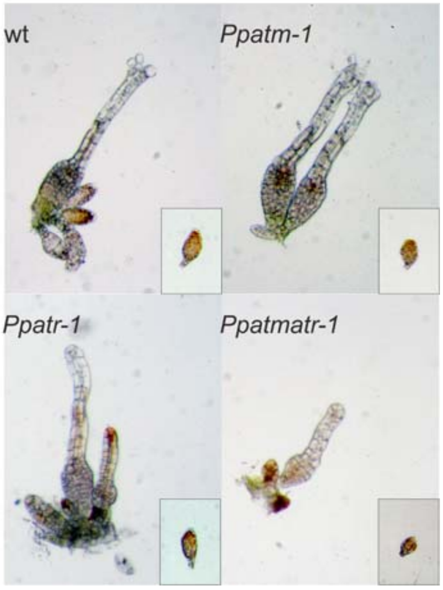

Figure S6: The mature gametophyte and reproductive structures: (A) Colonies were grown on minimal media at 15^0 C in an 8/16 hour day night cycle. The picture shows close-ups of wild type, *Ppatm-1*, *Ppatr-1* and *Ppatmatr-1* colonies at an age of four months. In wild type and *Ppatm-1*, almost all gametophores carry mature spore capsules at their tips while those are not present on *Ppatr-1* and *Ppatmatr-1* gametophores. **(B)** The pictures show the gametangia (reproductive structures) formed in wild type, *Ppatm-1*, *Ppatr-1* and *Ppatmatr-1*. Archegonia, the female organs are shown in the centre and the antheridia in the inset. Gametangia develop normally in *Ppatm-1*. Archegonia are abnormal in *Ppatr-1* or have degenerated shortly after fertilisation while antheridia appear normal. In *Ppatmatr-1*, both structures appear abnormal.

Supplemental tables:

Table S1

	Gene	human	yeast	*A. thaliana*	*P. patens*
checkpoint, regulators and signaling					
1	ATM/Tel1	+	+	+	Pp1s135_65V6.1
2	ATR/Mec1	+	+	+	Pp1s77_262V6.1
3	ATRIP	+		+	Pp1s60_281V6.1
4	ATRX	+		+	Pp1s34_370V6.1
5	BARD1	+		+	
6	BRCA1	+		+	
7	BRCA2/FANCD1	+		+	
8	BRCC36	+		+	Pp1s54_11V6.2
9	CHK1	+	+		
10	CHK2/Rad53	+	+		
11	DDC1		+		
12	DUN1		+		
13	hRAD1 /REC1	+		+	Pp1s67_171V6.1
14	HUG1	+	+		
15	Hus1	+	+	+	Pp1s59_63V6.1
16	LCD1		+		
17	MAPK family	+	+	+	Pp1s207_63V6.1, Pp1s138_117V6.1
18	MDC1	+			
19	MEC3		+		
20	P53	+			
21	PARP1	+		+	Pp1s114_181V6.1
22	PARP2	+		+	Pp1s324_39V6.1
23	PARP3	+		+	Pp1s59_305V6.1
24	PMYT1	+			Pp1s207_100V6.1
25	RAD17	+	+	+	Pp1s184_70V6.1
26	RAD24		+		
27	RAD9A/Sprad9	+		+	Pp1s130_202V6.1, Pp1s124_86V6.2
28	Rad9		+		
29	SOG1			+	Pp1s251_11V6.1
30	TP53B	+			
31	WEE1	+	+	+	Pp1s197_56V6.1
homologous recombination and meiosis					
32	BLM/RECQ2/ RECQL3/SGS1	+	+	+	Pp1s243_58V6.1
33	CTIP/RBBP8/COM1	+	+	+	Pp1s242_87V6.1
34	DMC1	+	+	+	Pp1s9_248V6.1
35	GEN1/YEN1	+	+	+	Pp1s391_28V6.1
36	HOP1		+		
37	HOP2	+	+	+	Pp1s335_13V6.1

38	INO80	+	+	+	Pp1s304_7V6.1, Pp1s45_2V6.1
39	MND1	+	+	+	Pp1s41_172V6.1
40	MRE11	+	+	+	Pp1s18_235V6.1
41	MUS81	+	+	+	Pp1s15_297V6.1
42	NBN/NBS/nimbrin	+		+	Pp1s219_52V6.1
43	PAXIP1	+		+	Pp1s97_25V6.1, Pp1s160_107V6.1, Pp1s35_92V6.1, Pp1s232_74V6.1
44	R51A1/RAD51AP1	+			
45	RA51B/RAD51B	+		+	Pp1s129_197V6.1
46	RA51C/RAD51C	+	+	+	Pp1s236_47V6.1
47	RA51D/RAD51D	+		+	Pp1s137_214V6.1
48	RA54B/RAD54B	+			Pp1s212_41V6.1
49	RAD5	+	+	+	Pp1s73_179V6.1, Pp1s41_174V6.1, Pp1s14_367V6.1
50	RAD50	+	+	+	Pp1s51_220V6.1
51	RAD51/ PpRAD51A PpRAD51B	+	+	+	Pp1s31_236V6.1, Pp1s42_140V6.1
52	RAD52	+	+		
53	RAD54	+	+	+	Pp1s341_67V6.1, Pp1s236_78V6.1, Pp1s212_41V6.1
54	RAD55		+		
55	RAD59		+		
56	RDH54/TID1		+		
57	REC102		+		
58	REC104		+		
59	REC8	+	+	+	Pp1s351_7V6.1
60	RecQ1	+		+	Wiedemann et al 2018
61	RecQ4	+		+	Wiedemann et al 2018
62	RECQ5	+		+	Wiedemann et al 2018
63	RED1		+		
64	RMI1	+	+	+	Pp1s201_114V6.1
65	RMI2	+		+	Pp1s159_85V6.1
66	RQSIM/AtRecQsim			+	Pp1s222_3V6.1, Pp1s152_88V6.1
67	RTEL1	+		+	Pp1s3_567V6.1
68	SGO1/Shugoshin 1		+		
69	SPO11	+	+	+	Pp1s62_130V6.2, Pp1s14_84V6.1, Pp1s248_24V6.1
70	SRS2		+	+	Pp1s21_400V6.1
71	SYCP1	+		+	
72	SYCP2	+		+	
73	SYCP3	+			
74	TOP3A	+	+	+	Pp1s475_4V6.1
75	TOP3B	+		+	Pp1s474_3V6.1

76	WRIP1	+	+	+	Pp1s16_272V6.1, Pp1s455_6V6.1, Pp1s97_156V6.1
77	WRN	+			Pp1s128_34V6.1
78	WRX (TAIR)			+	Pp1s246_95V6.1, Pp1s135_47V6.1, Pp1s4_355V6.1
79	XRCC2	+		+	Pp1s45_271V6.1
80	XRCC3	+		+	
81	XRS2		+		
82	ZIP1		+		
general functions and crosslink repair					
83	DCR1A/PSO2/SNM1	+	+	+	Pp1s377_22V6.1, Pp1s120_12V6.1
84	DCR1B	+		+	Pp1s68_3V6.1, Pp1s68_4V6.1
85	DDX11	+			Pp1s370_33V6.1
86	DNLI1	+	+	+	Pp1s223_54V6.2
87	DNLI3	+			
88	DNLI4	+	+	+	Pp1s150_94V6.1, Pp1s150_94V6.2
89	FANCA	+			
90	FANCB	+			
91	FANCC	+			
92	FANCD2	+		+	Pp1s204_101V6.1
93	FANCE	+			
94	FANCF	+			
95	FANCG	+			
96	FANCJ /BRIP1/BACH1	+		+	Pp1s95_66V6.1
97	FANCL	+		+	Pp1s156_74V6.1
98	FANCM	+	+	+	Pp1s9_477V6.1
99	LIF1		+		
100	PIF1	+	+	+	Pp1s152_136V6.1, Pp1s300_57V6.1
101	PNKP	+		+	Pp1s240_31V6.1
102	RAD21	+	+	+	Pp1s77_167V6.2, Pp1s77_195V6.2, Pp1s351_7V6.1
103	RNF4	+		+	Pp1s491_21V6.1, Pp1s121_28V6.2, Pp1s3_270V6.1, Pp1s369_26V6.1
104	RUVBL1	+	+	+	Pp1s4_73V6.1, Pp1s40_80V6.1, Pp1s40_79V6.1
105	RUVBL2	+	+	+	Pp1s255_64V6.1, Pp1s402_30V6.1, Pp1s88_18V6.1
106	SCC2	+	+	+	Pp1s104_102V6.1
107	SM1L2/SMC1B	+			
108	SMC1A	+	+	+	Pp1s91_43V6.1
109	SMC2	+	+	+	Pp1s52_57V6.1

110	SMC3	+	+	+	Pp1s410_17V6.1
111	SMC5	+	+	+	Pp1s274_85V6.1
112	SMC6	+	+	+	Pp1s61_278V6.1
113	STAG1	+	+	+	Pp1s199_157V6.1
114	STAG2	+			
115	STAG3	+			
116	TOF1		+		
117	TOPB1	+		+	Pp1s1_250V6.1
non-homologous end-joining					
118	DCR1C/ARTEMIS	+			
119	KU70/XRCC6	+	+	+	Pp1s299_4V6.1
120	KU80/XRCC5	+	+	+	Pp1s121_27V6.1
121	NHEJ1/XLF/ CERNUNNOS	+			
122	PRKDC/DNA-PKcs	+			Pp1s78_226V6.1
123	XRCC1	+		+	Pp1s224_52V6.1, Pp1s85_35V6.1
124	XRCC4	+		+	Pp1s34_261V6.1, Pp1s147_88V6.1
mismatch repair					
125	DIN7		+		
126	EME1	+		+	Pp1s72_302V6.1
127	EXO1	+	+	+	Pp1s10_231V6.2, Pp1s212_68V6.2
128	MLH1	+	+	+	Pp1s58_199V6.1
129	MLH2		+		
130	MLH3	+	+	+	Pp1s5_400V6.1
131	MSH1		+		
132	MSH2	+	+	+	Pp1s251_77V6.1
133	MSH3	+	+	+	Pp1s30_339V6.1
134	MSH4	+	+	+	Pp1s226_85V6.1
135	MSH5	+	+	+	Pp1s84_88V6.2
136	MSH6	+	+	+	Pp1s152_6V6.1, Pp1s90_86V6.1
137	Muts		+		Pp1s3_417V6.1
138	MUTYH	+		+	Pp1s151_27V6.1
139	PMS1/PMS2	+	+	+	Pp1s474_7V6.1
nucleotide excision, base excision and UV repair					
140	DDB1	+		+	Pp1s458_4V6.1, Pp1s203_55V6.1
141	DDB2	+		+	Pp1s114_132V6.1
142	ERCC1/Rad10	+	+	+	Pp1s117_170V6.1, Pp1s117_181V6.1
143	ERCC2/XPD	+	+	+	Pp1s145_26V6.3
144	ERCC3/Rad25/XPB	+	+	+	Pp1s177_124V6.1
145	ERCC5/RAD2	+	+	+	Pp1s31_24V6.1
146	ERCC6/rad26	+	+	+	Pp1s66_144V6.1, Pp1s34_212V6.1, Pp1s84_259V6.1, Pp1s155_61V6.3
147	RAD16		+	+	Pp1s3_639V6.1, Pp1s132_19V6.1

148	RAD18	+	+		
149	RAD23	+	+	+	Pp1s58_148V6.1, Pp1s286_52V6.1, Pp1s3_105V6.1, Pp1s3_98V6.1
150	RAD27/FEN1	+	+	+	Pp1s456_8V6.1, Pp1s39_160V6.2
151	RAD6/UBC2	+	+	+	Pp1s91_88V6.1, Pp1s91_87V6.1, Pp1s219_106V6.1
152	RAD7		+	+	
153	SYF1	+	+	+	Pp1s139_28V6.1
154	XPA/RAD14	+	+		
155	XPC/RAD4	+	+	+	Pp1s12_235V6.1
156	XPF/ERCC4/RAD1	+	+	+	Pp1s3_646V6.1
ATM and ATR interactors					
157	4EBP1/4E-BP1	+			
158	AATF (Che1)	+		+	Pp1s186_46V6.1
159	ABL1	+			
160	AKT1/PKB	+			
161	BID	+			
162	Cdc5l	+	+	+	Pp1s641_1V6.1
163	CHD family	+		+	Pp1s33_329V6.1, Pp1s235_76V6.1
164	CLSPN/Claspin	+			
165	CREB1	+			
166	E2F	+		+	Pp1s22_60V6.1, Pp1s38_356V6.1, Pp1s364_42V6.1, Pp1s97_96V6.1
167	H2A	+		+	Pp1s55_112V6.1, Pp1s452_4V6.1, Pp1s188_35V6.1
168	HDAC1	+	+	+	Pp1s223_52V6.1, Pp1s351_29V6.1, Pp1s180_68V6.1
169	HDAC2	+		+	
170	IKBA	+			
171	MCA3 (p18)	+			
172	MCM2	+	+	+	Pp1s28_266V6.1
173	MCM3	+	+	+	Pp1s9_156V6.1
174	MCM7	+	+	+	Pp1s31_86V6.1
175	MDM2	+			
176	MDM4	+			
177	PTPA	+	+	+	Pp1s111_153V6.1, Pp1s226_56V6.1
178	RENT1 (UPF1)	+	+	+	Pp1s10_103V6.1, Pp1s44_135V6.1
179	RFA1 (RPA)	+	+	+	Pp1s222_133V6.1, Pp1s192_40V6.1, Pp1s257_1V6.1

180	RFA2 (RPA)	+	+	+	Pp1s357_53V6.1
181	SOSB1/hSSB1	+		+	Pp1s112_133V6.1
182	SP1/TSFP1	+			
183	STRAP	+		+	Pp1s217_52V6.1, Pp1s25_22V6.1, Pp1s1020_4V6.1
184	TERF family	+		+	Pp1s74_197V6.1, Pp1s114_137V6.1, Pp1s176_113V6.1, Pp1s1_349V6.1, Pp1s176_88V6.1, Pp1s260_3V6.1, Pp1s49_258V6.1, Pp1s152_10V6.1
185	TIF1B	+			
186	TRIM1	+			

Table S1: Conservation of DNA damage repair-related genes between human, yeast and *A. thaliana* and the corresponding gene set in *P. patens*. The table lists genes from known sets in human and yeast and the occurrence of corresponding orthologues in human, yeast, *A. thaliana* and *P. patens* as obtained by BLAST analyses with corresponding protein sequences. The presence in human, yeast and *A. thaliana* is indicated by “+”, for *P. patens* the corresponding gene models are listed. Gene names are in UniProt nomenclature throughout and listed in the order human/synonym/yeast/fission yeast/*A. thaliana*. Additional synonyms are occasionally included. A conversion table of the v1.6 to the v3.3 *P. patens* genome annotation is available at http://plantco.de/research.php.

Table S2

P. patens gene model	Gene (UniProt nomenclature)	Bleo 0.3 1h / Bleo 0 1h	Bleo 3.0 1h / Bleo 0 1h	Bleo 0.3 1h - Bleo 0 3h / Bleo 0 1h
checkpoints, regulators and signalling				
Pp1s135_65V6.1	ATM	1.64	1.28	1.54
Pp1s60_281V6.1	ATRIP	0.64	0.51	0.65
Pp1s34_370V6.1	ATRX	9.76	4.91	7.49
Pp1s67_171V6.1	hRAD1 /REC1	1.48	1.98	4.55
Pp1s59_63V6.1	Hus1	7.10	5.95	10.68
Pp1s184_70V6.1	RAD17	2.37	3.21	8.43
Pp1s130_202V6.1	RAD9A/Sprad9 (1)	6.39	9.41	44.85
Pp1s251_11V6.1	ANAC008/SOG1	0.84	0.61	1.27
Pp1s207_63V6.1	MAPK family (1)	0.49	0.63	0.12
Pp1s138_117V6.1	MAPK family (2)	0.47	0.45	0.18
Pp1s207_100V6.1	PMYT1	0.97	0.84	1.21
Pp1s197_56V6.1	WEE1	2.22	1.70	6.18
Pp1s324_39V6.1	PARP2	2.98	4.59	4.97
homologous recombination and meiosis				
Pp1s93_66V6.1	RecQ1/RQL2 Arath	0.44	0.65	1.04
Pp1s152_88V6.1	RQSIM	0.73	0.59	1.86
Pp1s201_114V6.1	RMI1	0.59	0.87	0.98
Pp1s159_85V6.1	RMI2	1.06	0.80	0.76
Pp1s15_297V6.1	MUS81	0.38	0.52	0.37
Pp1s219_52V6.1	NBN	0.97	0.91	1.61
Pp1s236_47V6.1	RA51C	1.04	1.70	2.25
Pp1s212_41V6.1	RA54B	1.90	3.40	8.67
Pp1s42_140V6.1	PpRAD51B	0.89	4.08	3.37
Pp1s31_236V6.1	PpRAD51A	3.53	7.20	10.48
Pp1s341_67V6.1	RAD54 (1)	2.80	2.81	7.33
Pp1s236_78V6.1	RAD54 (2)	2.66	3.68	3.09
Pp1s255_64V6.1	RUVB2	2.92	1.78	2.25
Pp1s16_272V6.1	WRIP1 (1)	1.10	1.04	3.46
Pp1s455_6V6.1	WRIP1 (2)	0.43	0.62	0.43
Pp1s135_47V6.1	WRX (1)	1.34	0.84	1.35
Pp1s4_355V6.1	WRX (2)	1.71	0.79	1.65
Pp1s45_271V6.1	XRCC2	0.55	0.30	0.49
Pp1s97_25V6.1	PaxIP1 (1)	1.23	1.12	1.46
Pp1s160_107V6.1	PaxIP1 (2)	0.25	1.78	1.12
Pp1s35_92V6.1	PaxIP1 (3)	4.90	3.50	1.47
Pp1s232_74V6.1	PaxIP1 (4)	0.61	0.83	0.50
Pp1s335_13V6.1	HOP2	0.83	0.96	1.93

Pp1s41_172V6.1	MND1	2.92	1.94	2.89
Pp1s62_130V6.1	SPO11	0.87	0.41	0.87
general functions and cross link repair				
Pp1s370_33V6.1	DDX11	0.73	0.88	0.87
Pp1s223_54V6.2	DNL1	1.56	2.57	5.15
Pp1s240_31V6.1	PNKP	3.11	4.37	11.08
Pp1s91_43V6.1	SMC1A	0.74	0.76	1.12
Pp1s274_85V6.1	SMC5	0.83	0.90	1.57
Pp1s77_195V6.2	RAD21	1.18	1.42	0.19
Pp1s491_21V6.1	RNF4 (1)	1.30	1.22	1.21
Pp1s121_28V6.2	RNF4 (2)	1.37	1.08	0.92
Pp1s3_270V6.1	RNF4 (3)	1.37	1.25	1.67
Pp1s300_57V6.1	PIF1	2.13	1.81	4.72
Pp1s104_102V6.1	SCC2	0.84	0.57	0.56
Pp1s68_122V6.2	SIR2 (1)	1.02	0.58	1.15
Pp1s272_33V6.1	SIR2 (2)	0.55	0.57	0.91
Pp1s15_90V6.2	SIR2 (3)	0.82	0.57	0.65
Pp1s204_101V6.1	FACD2	0.47	0.07	0.86
Pp1s156_74V6.1	FANCL	0.77	0.69	1.05
non-homologous end-joining				
Pp1s299_4V6.1	KU70/xrcc6	1.67	3.14	16.70
Pp1s121_27V6.1	KU80/XRCC5	1.28	1.79	4.38
Pp1s78_226V6.1	PRKDC	0.82	0.49	0.32
Pp1s224_52V6.1	XRCC1	2.66	3.40	3.37
Pp1s147_88V6.1	XRCC4	9.32	19.27	126.46
mismatch repair				
Pp1s72_302V6.1	EME1	0.68	0.40	0.48
Pp1s58_199V6.1	MLH1	0.58	0.52	1.58
Pp1s5_400V6.1	MLH3	1.33	0.57	2.62
Pp1s3_417V6.1	Muts	0.59	1.32	0.94
Pp1s251_77V6.1	MSH2	0.96	1.11	1.67
Pp1s30_339V6.1	MSH3	1.63	0.87	0.90
Pp1s84_88V6.1	MSH5	0.63	0.60	0.66
Pp1s474_7V6.1	PMS1/PMS2	0.63	0.92	1.45
Pp1s10_231V6.2	EXO1	0.89	0.58	0.98
nucleotide excsision, base excision and UV repair				
Pp1s117_170V6.1	ERCC1/Rad10 (1)	0.94	1.46	1.35
Pp1s145_26V6.3	ERCC2/XPD	1.05	1.73	3.24
Pp1s177_124V6.1	ERCC3/Rad25/XPB	0.85	1.37	2.33
Pp1s3_646V6.1	XPF/ERCC4/RAD1	1.26	1.98	4.03
Pp1s31_24V6.1	ERCC5/RAD2	0.23	0.39	0.64

Pp1s66_144V6.1	ERCC6/rad26 (1)	2.03	1.66	2.17
Pp1s155_61V6.3	ERCC6/rad26 (2)	0.33	0.71	0.07
Pp1s139_28V6.1	SYF1	0.62	0.60	1.02
Pp1s3_639V6.1	RAD16 (1)	1.51	1.68	1.12
Pp1s132_19V6.1	RAD16 (2)	1.98	2.01	2.16
Pp1s58_148V6.1	RAD23 (1)	0.81	1.01	0.57
Pp1s286_52V6.1	RAD23 (2)	1.29	1.70	1.92
Pp1s3_105V6.1	RAD23 (3)	1.78	0.89	1.94
Pp1s456_8V6.1	RAD27/FEN1 (1)	0.78	0.85	0.70
Pp1s39_160V6.1	RAD27/FEN1 (2)	2.66	1.51	5.25
Pp1s91_87V6.1	RAD6/UBC2 (1)	0.94	0.90	0.81
Pp1s219_106V6.1	RAD6/UBC2 (2)	1.22	1.25	1.29
Pp1s458_4V6.1	DDB1 (1)	0.94	0.79	0.98
Pp1s203_55V6.1	DDB1 (2)	1.68	0.48	1.36
Pp1s114_132V6.1	DDB2	1.91	1.90	3.99
ATM and ATR interactors				
Pp1s33_329V6.1	CHD family (1)	0.61	0.54	0.53
Pp1s235_76V6.1	CHD family (2)	0.89	0.88	0.53
Pp1s22_60V6.1	E2F1	0.74	0.70	2.19
Pp1s55_112V6.1	H2A(1)	1.18	0.66	0.45
Pp1s452_4V6.1	H2A (2)	2.73	0.74	1.56
Pp1s223_52V6.1	HDAC1 (1)	0.81	0.47	0.56
Pp1s351_29V6.1	HDAC1 (2)	0.71	0.91	0.93
Pp1s180_68V6.1	HDAC1 (3)	1.34	1.66	0.92
Pp1s31_86V6.1	MCM7	1.69	1.36	0.67
Pp1s226_56V6.1	PTPA (1)	1.19	1.13	2.02
Pp1s111_153V6.1	PTPA (2)	1.42	3.17	2.47
Pp1s10_103V6.1	RENT1 (UPF1) (1)	2.04	2.95	1.80
Pp1s222_133V6.1	RFA1 (RPA)	3.35	3.40	3.00
Pp1s357_53V6.1	RFA2 (RPA)	1.04	1.18	1.05
Pp1s112_133V6.1	SOSB1 (SSB1)	0.55	0.50	0.93
Pp1s217_52V6.1	STRAP (1)	0.42	0.07	0.33
Pp1s25_22V6.1	STRAP (2)	0.86	0.81	1.14
Pp1s114_137V6.1	TERF family (1)	1.04	0.85	0.69
Pp1s176_113V6.2	TERF family (2)	1.53	0.97	1.22
Pp1s1_349V6.1	TERF family (3)	0.55	0.88	0.80

Table S2: The early response of DNA damage repair-related genes to bleomycin-induced DNA damage. The differential expression profiles obtained for the treatment and mock treatment, respectively, of wild type with lethal (3.0 u/l) and sub-lethal (0.3 u/l) concentrations of bleomycin for 1 hour and the following 3 hour recovery period are shown for the set of DNA

damage repair-related genes. The fold-change ratios are: Bleo 0.3 1h / Bleo 0 1h, 1 h 0.3 u/l treatment (T1/0.3) divided by mock treatment for 1 h (T1/0); Bleo 3.0 1h / Bleo 0 1h, 1 h 3.0 u/l treatment (T1/3.0) divided by mock treatment for 1 h (T1/0); Bleo 0.3 1h – Bleo 0 3h /Bleo 0 1h, 1 hour 0.3 u/l treatment followed by 3 h recovery (T4/0.3) divided by mock treatment for 1 h (T1/0). The nomenclature is as in table S1 and table 1.
